# ﻿A review of the genus *Pempheris* (Teleostei, Pempheridae) found in Japan and Taiwan

**DOI:** 10.3897/zookeys.1220.126762

**Published:** 2024-12-09

**Authors:** Keita Koeda, Manabu Bessho-Uehara

**Affiliations:** 1 Faculty of Science, University of the Ryukyus, 1 Senbaru, Nishihara, Okinawa 903-0213, Japan; 2 The Frontier Research Institute for Interdisciplinary Sciences, Tohoku University, Sendai, Japan; 3 Graduate School of Life Sciences, Tohoku University, Sendai, Japan

**Keywords:** Distribution, morphology, *Pempherissasakii*, *Pempherisxanthoptera*, sweepers, taxonomy

## Abstract

Species of the genus *Pempheris* Cuvier, 1829 (Pempheridae) from Japan and Taiwan are taxonomically reviewed based on morphology supported by molecular phylogenetic analysis. Ten species are recognized from these countries: *Pempherisadusta* Bleeker, 1855, *Pempherisfamilia* Koeda & Motomura, 2017, *Pempherisjaponica* Döderlein, 1883, *Pempherisnyctereutes* Jordan & Evermann, 1902, *Pempherisoualensis* Cuvier, 1831, *Pempherissasakii* Jordan & Hubbs, 1925, *Pempherisschwenkii* Bleeker, 1877, *Pempherisufuagari* Koeda, Yoshino & Tachihara, 2013, *Pempherisvanicolensis* Cuvier, 1831, *Pempherisxanthoptera* Tominaga, 1963. Nine of them are distributed in Japan, and five of them in Taiwan. *Pempherissasakii* and *P.xanthoptera*, nominal species that have been regarded as invalid are revalidated, redescribed with diagnoses based on examinations of the holotypes and the specimens collected from Japan. *Pempherissasakii* is morphologically similar to *P.nyctereutes* and has been thought to be a senior synonym of the latter, but the comparison of the holotypes and non-types of both species revealed that the former species is distinguishable from the latter species in having fewer counts of body scales, also genetically supported with a 3.1% mitochondrial DNA sequence divergence. *Pempherisxanthoptera* is similar to *P.schwenkii*, but the coloration of their caudal fins is different, and the genetic analysis supported the difference. The distributions of all species of the genus *Pempheris* in Japanese waters are also described, based on the specimen localities from literature and new material herein.

## ﻿Introduction

The family Pempheridae, also known as sweepers, is a group of nocturnal fish widely distributed in the Indo-Pacific and western Atlantic Ocean. This family is currently divided into two genera: *Parapriacanthus* Steindachner, 1870 and *Pempheris* Cuvier, 1829. The latter is characterized by having anal-fin soft rays numbering 30–45, the anal-fin base covered with scales and longer than 40% of the standard length, the lateral line extending onto the posterior margin of the caudal fin, and the first interhaemal angled toward the posterior end of the dorsal-fin base (Tominaga 1963). The genus *Pempheris* was first proposed by Cuvier (1829) for *Pempheristouea* Cuvier, 1829, which is presently recognized as a junior synonym of *Pempheriscompressa* (Shaw, 1790) (Tominaga 1968). In total, 84 nominal species have been described for the genus to date (Fricke et al. 2024), with almost half of them newly described from the western Indian Ocean from 2014 to 2015 (e.g., Randall and Victor 2014; Randall and Victor 2015). Because the validity of these Indian Ocean species needs to be reassessed, the taxonomy of the whole genus has long been confused due to difficulties in identifying its species.

The taxonomy of the family Pempheridae in Japanese waters was reviewed by Tominaga (1963) based on a comparison of morphology, and he recognized four species of the genus *Pempheris* from Japan. Since then, two new species have been described: *Pempherisfamilia* Koeda & Motomura, 2017 and *Pempherisufuagari* Koeda, Yoshino & Tachihara, 2013, and two new-to-Japan species were reported, *Pempherisoualensis* Cuvier, 1831 and *Pempherisvanicolensis* Cuvier, 1831, from the Japanese waters (Koeda et al. 2010a, b, 2013a; Koeda and Motomura 2017a). However, the validity of *Pempherissasakii* Jordan & Hubbs, 1925 and *Pempherisxanthoptera* Tominaga, 1963, both described from Japan, have never been evaluated. Therefore, the present study conducted a direct comparison of the morphology, including of the type specimens of *Pempherisnyctereutes* Jordan & Evermann, 1902 and *Pempherisschwenkii* Bleeker, 1877. Although many species of the genus commonly distributed in Japan are also found in Taiwan, the taxonomy of the genus in Taiwan has never been reviewed, and misidentifications are occasionally observed in the Taiwanese literature. Here, we reviewed the species of the genus *Pempheris* that occurred in Japan and Taiwan with detailed descriptions, based on the type specimens and large numbers of non-types, providing diagnoses, identification keys, and distributional ranges. Additionally, the published literature which relates to the genus *Pempheris* of Japan and Taiwan was re-examined and corrected as much as possible.

## ﻿Materials and methods

Preserved materials examined in the present study including large numbers of specimens collected by KK are listed in Suppl. material 1. Comparative materials of the type specimens of species of the genus *Pempheris* are listed in Koeda et al. (2013a, 2014) and Koeda and Motomura (2017a). Counts and measurements followed Koeda et al. (2014). All measurements were made on the left side when possible, using digital calipers and rounded to the nearest 0.1 mm. Standard and head lengths are abbreviated as **SL** and **HL**, respectively. Osteological characters, including vertebral counts, were observed from radiographs. The descriptions of general morphology shared by species of the genus are not repeated here. Data of type specimens are given in parentheses. The distribution maps were made based on the specimens examined and collected by KK, quality underwater photographs, and literature records with a good illustration and/or sufficient diagnostic information to provide for positive identification. Photographs were taken by KK except when a credit line is given. Synonym lists for each species are shown only for the related references for Japan and Taiwan and the original descriptions. Institutional codes used in this study follow Fricke et al. (2024) with an addition: University of the Ryukyus, Ichthyological Laboratory (**URIL**).

The nucleotide sequences of mitochondrial 16S ribosomal RNA (*16S*) and cytochrome oxidase I (COI) were analyzed to infer phylogenetic relationships. DNA was extracted from ethanol-fixed specimens and the sequences of *16S* and *COI* were obtained as described by Koeda et al. (2014). We concatenated and aligned the obtained *16S* and *COI* sequences using MAFFT alignment (v. 7.490) (Katoh and Standley 2013) with default settings in Geneious Prime software (v. 2023.2.1) (Biomatters). Uncorrected pairwise distances (p-distances) among the sequences of different specimens were estimated using Geneious Prime software. Phylogenetic relationships were inferred using maximum-likelihood (ML) inference, neighbor-joining (NJ), and Bayesian inference (BI) methods. The ML tree was reconstructed using IQ-TREE 1.6.12 (Trifinopoulos et al. 2016) with a partitioned model for *16S* (sites 1–481) and *COI* (sites 482–837). The best-fit substitution model was chosen by ModelFinder with “Auto” option which automatically selects the best-fit model for each partition. We performed the Shimodaira–Hasegawa-like approximate likelihood ratio test (SH-aLRT) 1,000 times to assess the nodal support (Guindon et al. 2010). The NJ tree was reconstructed using the Tamura-Nei model, and a consensus tree was generated by resampling 10,000 replicates of bootstrap analysis. The BI tree was constructed with MrBayes 3.2.6 (Huelsenbeck and Ronquist 2001), using the GTR substitution model and the invgamma rate variation model. Four independent Markov chain Monte Carlo (MCMC) runs were conducted for 2,100,000 generations, subsampling trees every 200 cycles, with the initial 100,000 trees discarded as burn-in. The majority rule consensus of the remaining trees was used to determine clade posterior probabilities.

## ﻿Taxonomic account

### ﻿Genus Pempheris

Taxon classificationAnimaliaPerciformesPempheridae

Cuvier, 1829

C01F69A9-ECF7-5803-9B27-B7544A769688

Pempheris Cuvier, 1829: 195 [type species: Pempheristouea = Kurtusargenteus Bloch & Schneider, 1801 = Sparus?compressus (Shaw, 1790): junior synonym of Pempheriscompressa (Shaw, 1790)].
Priacanthopsis Fowler, 1906: 122 (type species: Pempherismulleri: junior synonym of Pempherisschomburgki Müller & Troschel, 1848)].
Catalufa Snyder, 1911: 528 (type species: Catalufaumbra: junior synonym of Pempherisjaponica Döderlein, 1883).
Liopempheris Ogilby, 1913: 61 (type species: Pempherismultiradiatus Klunzinger, 1879).

#### Description.

Body shape oval, strongly compressed laterally; body deep, deepest at or near origin of dorsal fin; dorsal outline of head nearly straight or generally curved from snout to origin of dorsal fin; ventral outline of body generally curved to origin of pelvic fin; body depth rapidly decreases at posterior half of body; depth of caudal peduncle < 1/4 of maximum body depth.

Eye large; snout very short; interorbital space slightly convex or flat; two nostrils located just anterior to anterior margin of eye. Mouth large, strongly oblique; lower jaw slightly project beyond upper jaw; villiform teeth on jaws; tip of tongue free from floor of mouth. Lips thin. Gill opening large; outer margin of opercle and preopercle smooth. Gill membranes on left and right sides separate, free from isthmus. Gill rakers long, 6–13 (upper)+17–28 (lower) on first gill arch.

Body and head almost fully covered by strongly or weakly ctenoid scales except for lips and anterior to eye; ~ 1/3 of basal part of anal fin covered with small scales. Lateral line starts from uppermost position of opercle, generally follows dorsal outer margin, through middle of caudal peduncle, and extending to middle of posterior end of caudal fin. Anus slit-like, located just anterior to anal fin. Light organ present in some species (absent in species distributed in the Northern Hemisphere).

Vertebral counts 10+15 (abdominal + caudal), very rarely 10+16; predorsal interneurals 3; 4^th^ interneural supporting 1^st^ dorsal-fin spine, inserted between 2^nd^ and 3^rd^ vertebrae; last interneural with last dorsal ray inserted between neural spines of 11^th^ and 12^th^ vertebrae, or 12^th^ and 13^th^. First interhaemal supporting 1^st^ and 2^nd^ anal-fin spine, inserted in front of haemal spine of 11^th^ vertebra, and pointing to posterior end of dorsal fin.

Dorsal fin single, triangular; its base short, shorter than longest ray; 5–7 spines, last longest; 8–13 soft rays, 1^st^ or 2^nd^ longest, rapidly shorter posteriorly. Anal fin low, its base very long, length longer than body depth; three spines, last longest; 24–49 soft rays, 1^st^ longest, gradually shorter posteriorly. Pectoral fin pointed posterodorsally; 15–20 rays, uppermost two rays unbranched, 3^rd^ or 4^th^ ray longest, shorter in lower; pectoral-fin length longer than length of longest dorsal-fin ray. Pelvic fin small, with one spine and five soft rays, 1^st^ longest; last ray not connected to body with membrane. Caudal fin triangular, weakly forked.

Body color uniformly silver, copper, or golden without distinct patterns except for *Pempherisornata* Mooi & Jubb, 1996 (not in the Northern Hemisphere) which has longitudinal golden stripes on body laterally.

#### Distribution.

Indo-Pacific Ocean: north to southern Japan, east to Easter Island (not including Hawaii Islands), south to Tasmania, west to South Africa, and the Red Sea (some species migrated from the Red Sea to the eastern part of Mediterranean); western Atlantic Ocean: north to Florida; south to Brazil (Mouneimne 1979; Golani and Ben-Tuvia 1986; Golani and Diamant 1991; Koeda et al. 2014).

#### Remarks.

This genus includes a large number of species, and the counts, measurements, and colorations are not very informative in distinguishing them from each other because of the interspecific uniformity and the intraspecific diversity of the results. This had led to significant taxonomic confusions, and the recent jumbled descriptions of abnormally high numbers of new species reported from the Indian Ocean have caused further misunderstandings in the taxonomy of the genus.

Fowler (1906), Snyder (1911), and Ogilby (1913) attempted to divide the genus *Pempheris* into two genera based on scale morphology. Tominaga (1968) described and compared the internal anatomy of many species of the genus and suggested that several species of genus *Pempheris* possess transitional characteristics to the genus *Parapriacanthus*. Therefore, he subdivided the genus *Pempheris* into seven groups. That work indicated that a systematic revision of the genus should be pursued. Although the authors of the present study are now revising the systematic taxonomy of the family Pempheridae on the basis of morphology and molecular approaches, the present classification of the genus *Pempheris* is tentatively used only for the species of Japan and Taiwan.

Molecular phylogenetic analysis using three methods (ML, NJ, and BI) showed consistent topology, except for the placement of *P.ufuagari*. In the ML and BI trees, *P.ufuagari* is positioned as a sister to a clade composed of *P.vanicolensis* and *P.oualensis* with low node supporting values. In contrast, the NJ tree places *P.ufuagari* as a sister to a clade composed of *P.vanicolensis* and *P.adusta*. All species analyzed in this study displayed monophyly with high supporting values on their respective nodes.

### ﻿Key to the species of genus *Pempheris* in Japan and Taiwan (with distributions in parentheses after the species name)

**Table:** 

1	12–15 scale rows above lateral line; scales on lateral body strongly ctenoid and adherent, with distinct basal and distal portions (Koeda et al. 2013a: fig. 2b); ventral surface of abdomen rounded, cross-sectional outline U-shaped; coracoid slightly expanded posteriorly; large ventral fenestra between coracoid and cleithrum	**2**
–	3½–10½ scale rows above lateral line; scales on lateral body weakly ctenoid and deciduous (Koeda et al. 2013a: fig. 2a), semicircular in shape; ventral surface of abdomen slightly or well keeled, cross-sectional outline V-shaped; coracoid enormously expanded posteriorly; very small ventral fenestra between coracoid and cleithrum	**3**
2	69–82 pored lateral-line scales; 12 or 13 scale rows above lateral line; 40–44 predorsal scales; 22–24 circumpeduncular scales; blackish blotch on the pectora-fin base absent or faint	***P.japonica*** (southern Japan, Izu Islands, western Japan Sea, rarely in Ryukyu Archipelago; southern Korea)
–	84–88 pored lateral-line scales; 14 or 15 scale rows above lateral line; 50–55 predorsal scales; 26 circumpeduncular scales; distinct pupil-sized blackish blotch on the pectoral-fin base present	***P.familia*** (Ogasawara Islands)
3	67–81 pored lateral-line scales; 8½–9½ scale rows above lateral line; 19–27 scale rows below lateral line; snout pointed; scales on ventral and pored lateral-line scales strongly ctenoid; body brownish with golden reflection; lateral line distinctly whitish	**4**
–	44–71 pored lateral-line scales; 3½–7½ scale rows above lateral line; 10–18 scale rows below lateral line; snout weakly pointed or rounded; scales on ventral and pored lateral-line scales weakly ctenoid; body pale brown to grey with golden, silver, or copper reflections; lateral line same as uniform color of body	**5**
4	67–77 pored lateral lateral-line scales, usually fewer than 73; 19–22 scale rows below lateral line; body brown, with golden reflection in fresh specimen	***P.sasakii*** (southern Japan, northern Ryukyu Archipelago)
–	72–81 pored lateral lateral-line scales, usually > 79; 23–28 scale rows below lateral line; body silver to dark brown in fresh specimen	***P.nyctereutes*** (Taiwan; Vietnam)
5	44–65 pored lateral lateral-line scales; 3½–6½ scale rows above lateral line; black blotch on pectoral-fin base absent; pectoral fin uniformly pink or bright yellow	**6**
–	51–71 pored lateral lateral-line scales; 4½–7½ scale rows above lateral line; black blotch on pectoral fin-base present; pectoral fin uniformly pink or upper half dusky	**8**
6	57–65 pored lateral lateral-line scales; 5½–6½ scale rows above lateral line; 12–15 scale rows below lateral line; body with silver reflection; pectoral fin bright yellow; outer margin of anal fin distinctly blackish	***P.vanicolensis*** (Ryukyu Archipelago, Taiwan; western Pacific)
–	44–54 pored lateral lateral-line scales; 3½–4½ (usually 3½) scale rows above lateral line; 10–12 scale rows below lateral line; body with golden or silver reflection; pectoral fin pink; outer margin of anal fin faint blackish or translucent	**7**
7	Posterior nostril usually oval, rounded; caudal fin bright yellow	***P.xanthoptera*** (southern Japan, Izu Islands, western Japan Sea, northern Ryukyu Archipelago, Ogasawara Islands; southern Korea)
–	Posterior nostril usually slit-like; caudal fin pink to brown	***P.schwenkii*** (southern Kyusyu, Ryukyu Archipelago; Taiwan; western Pacific)
8	51–62 pored lateral lateral-line scales; 4½–5½ scale rows above lateral line; black blotch on pectoral-fin base faint, usually on posterior 2/3 of its base	***P.adusta*** (southern Japan, Izu Islands, Ryukyu Archipelago, Daito Islands, Ogasawara Islands; Taiwan; western Pacific)
–	60–71 pored lateral lateral-line scales; 6½–7½ scale rows above lateral line; distinct black blotch covering entire pectoral-fin base present	**9**
9	Usually 7½ scale rows above lateral line; tooth band absent at outside of lips; dorsal and caudal fins bright yellow; pectoral fin uniformly pink; tip of dorsal fin black, but anterior margin not black; outer margin of anal fin distinctly blackish	***P.ufuagari*** (Daito Islands, Ogasawara Islands)
–	Usually 6½ scale rows above lateral line; tooth band present at outside of lips (in large individuals); dorsal and caudal fins brown; upper half of pectoral fin dusky; anterior margin to tip of dorsal fin black; outer margin of anal fin without black coloration	***P.oualensis*** (Ryukyu Archipelago, Daito Islands, Ogasawara Islands; Taiwan; Western Pacific)

### ﻿ Pempheris adusta

Taxon classificationAnimaliaPerciformesPempheridae

Bleeker, 1877

8B0BE0D4-EEBE-5719-BB8E-5E482A4A0975

**Figure 1.**
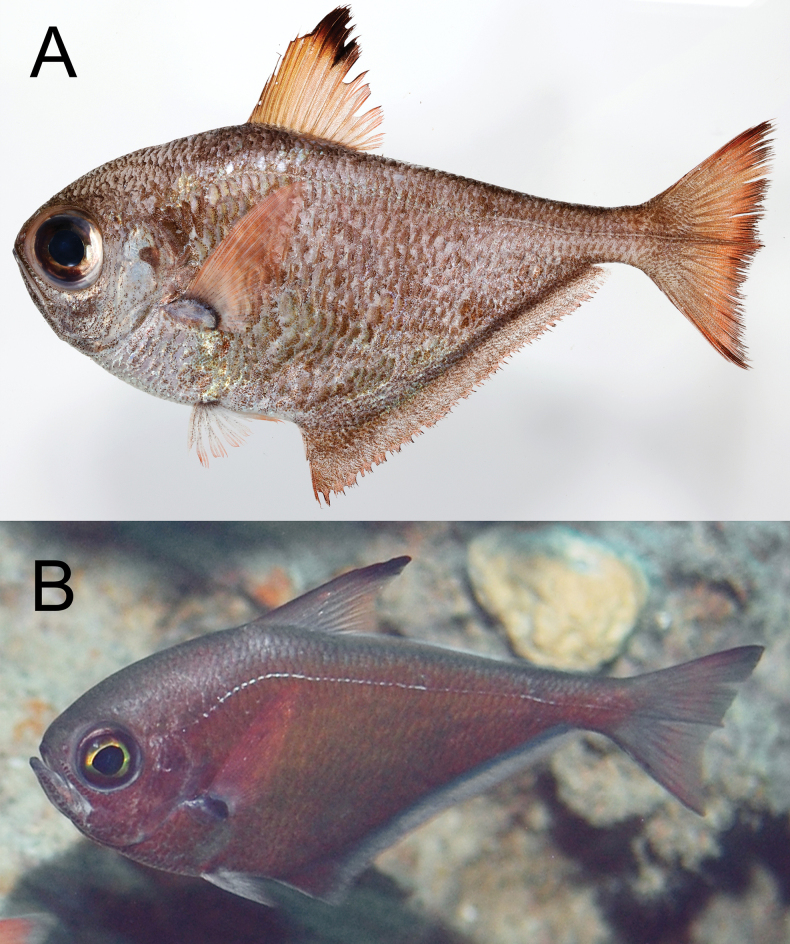
*Pempherisadusta***A** fresh specimen (ZUMT 62301, Nishidomari, Otsuki, Kochi, Japan) and **B** underwater photograph (Maeda, Onna, Okinawa-jima Island, Japan).

**Figure 2.**
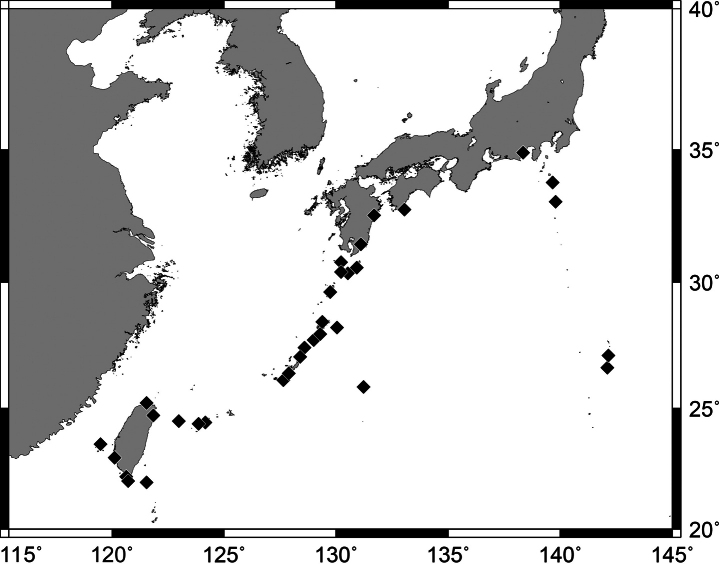
Distribution of *Pempherisadusta* based on the collection localities of specimens.

Figs 1 , 2 , Suppl. material 2 Standard Japanese name: Ryukyu-hatampo

Pempheris adusta Bleeker, 1877: 50, pl. 383, fig. 1 (type locality: Ambon Island, Molucca Islands, Indonesia); Koeda et al. 2013a: 235; Koeda et al. 2013b: 221, fig. 1; Koeda et al. 2013c: 123, fig. 1; Koeda et al. 2014: 303, fig. 1; Motomura and Matsuura 2014: 270, unnumbered figs; Koeda and Motomura 2015: 139, fig. 1; Koeda et al. 2015: 279; Kaburagi 2016: 98, upper fig. (without scientific name; shown as “Ryukyu-hatampo” in Japanese); Koeda et al. 2016a: 519; Koeda et al. 2016b: 50, fig. 224; Koeda et al. 2016c: 8, fig. 3-G; Koeda and Motomura 2017a; Koeda 2017a: 5, fig. 1 (middle fig.); Kimura et al. 2017: 119, fig. 5; Planning and Tourism Division of Kikai Town 2017: 4, unnumbered figs; Nakae et al. 2018: 266; Koeda 2018a: 193, unnumbered figs; Koeda 2018b: 298, unnumbered fig.; Koeda 2018c: 340, unnumbered figs; Mochida and Motomura 2018: 30; Koeda 2019: 926, unnumbered figs; Murase et al. 2019: 132, fig. 283; Fujiwara and Motomura 2020: 28; Koeda 2020a: 407, unnumbered figs; Koeda 2020b: 926, unnumbered figs; Motomura and Uehara 2020: 45; Murase et al. 2021: 166, fig. 339; Koeda et al. 2022: 5; Motomura 2023: 126.
Pempheris mangula (not Cuvier, 1829): Schmidt 1913: 121; Randall and Lim 2000: 622.
Pempheris oualensis (not Cuvier, 1831): Snyder 1912: 497; Okada 1938: 179; Okada and Matsubara 1938: 179; Aoyagi 1948: 49; Matsubara 1955: 590; Tominaga 1963: 289; Honda 1972: 72; Masuda et al. 1975 (in part): 199, pl. 33-D; Yoshino et al. 1975: 75; Hayashi 1984 (in part): 160, pl. 151-E; Shen 1984: 74, pl. 74, fig. 334-1; Shao et al. 1992: 177, unnumbered fig.; Shen 1993: 390, pl. 114 (fig. 1); Shao and Chen 1991: 162, unnumbered fig.; Chen et al. 1995 (probably in part): 25; Mochizuki 1995: 389; Yoshigou et al. 2001: 141; Chen 2003 (in part): 134; Ito 2009: 80, unnumbered fig.; Shao et al. 2008: 254; Chen et al. 2010: 265, fig. D; Chang et al 2011: 46; Shen and Wu 2011: 498, unnumbered fig; Shao et al. 2013 (in part): 163, unnumbered fig. (upper left); Chiang et al. 2014: 183, unnumbered fig.
Pempheris sp.: Uchida 1933: 218 (in part); Senou et al. 2006a: 77; Senou et al. 2007: 56; Hatooka 2002 (in part): 878; Senou et al. 2002: 212; Yoshino 2008: 211, unnumbered figs (lower two); Koeda et al. 2010a: 75; Motomura et al. 2010 (in part): 131; Koeda et al. 2012a: 71; Koeda et al. 2012b: 1086; Hatooka and Yagishita 2013 (in part): 984; Motomura et al. 2013: 169, unnumbered figs.
Pempheris vanicolensis (not Cuvier, 1831): Chen et al. (2010): 266, fig. B.; Shen and Wu 2011: 498, unnumbered fig.

#### Diagnosis.

Counts of holotype and non-types are given in Table 1 of Koeda et al. (2013b). Dorsal-fin spines 5 or 6, very rarely 5, soft rays 8–10, very rarely 8 or 10; anal-fin spines 3, soft rays 37–45, usually > 40; pectoral-fin rays 16–19, usually 17 or 18; pored lateral-line scales 51–62, usually > 54; scale rows above lateral line 4½–5½ (usually 4½); scale rows below lateral line 11–16, usually 12–14; predorsal scales 26–38; circumpeduncular scales 12–18, usually 16; gill rakers 7–10+20–23 = 28–32, usually 8–9+20–22 = 29–31; head length 26.3–31.8% SL; body depth 40.2–47.3% SL; eye diameter 36.0–47.1% HL; upper jaw length 48.1–57.1% HL; maximum 182.7 mm SL, usually < 160 mm SL; scales weakly ctenoid, deciduous, thin, semicircular in shape, far wider than long; body copper to brownish, whiter in nighttime; faint blackish blotch on pectoral-fin base; tip and/or anterior margin of dorsal fin blackish; blackish band on outer margin of anal fin usually absent; blackish or dusky band on posterior edge of caudal fin; narrow band of villiform teeth in jaws; abdomen cross-sectional outline V-shaped.

**Table 1.** Counts of *Pempherisnyctereutes* and *P.sasakii*.

	*P.nyctereutes*		*P.sasakii*	
	Holotype	Non-types	Holotype	Non-types
Number of individuals	1	18	1	47
Standard length (mm)	160.5	100.5–162.4	93.1	87.7–169.8
Dorsal fin rays	VI, 9	VI, 9	VI, 9	VI–VII, 9–10
Anal fin rays	III, 44	III, 42–44	III, 43	III, 40–46
Pectoral fin rays	19	18–20	19	17–20
Left pored lateral-line scales	79	72–81	72	67–78
Right pored lateral-line scales	77	74–82	73	66–78
Scale above lateral line	8 1/2	8 1/2–9 1/2	8 1/2	8 1/2–10 1/2
Scale rows below lateral line	23	23–28	22	19–22
Circumpeduncular scales	22	22–24	damaged	24
Gill rakers	8+20	8+19–20	8+19	7–9+19–22

#### Distribution.

Widely distributed in the western Pacific Ocean excepting small oceanic islands and atolls in central and southeastern Pacific. In Japanese waters, this species is known from Yaizu in Shizuoka Prefecture, Iburi and Otsuki in Kochi Prefecture, Nagasaki in Nagasaki Prefecture, Uchinoura Bay in Kagoshima Prefecture, Hachijo-jima islands in Izu Islands, Tanega-shima to Yonaguni-jima islands in the Ryukyu Archipelago, Minamidaito-jima Island in the Daito Islands, Miyake-jima and Hachijo-jima islands in the Izu Islands, Haha-shima and Chichi-jima islands in the Ogasawara Islands. In Taiwanese waters, this species is known from Daxi in Yilan County, Yeh Liu in New Taipei City, Sihhu in Yunlin County, Tainan County, Checheng County, Hengchung, and Kenting in Pingtung County, Fugang in Taitung County, Lyudao, Lanyu, Xiao Liuqiu, and Penghu (Fig. 2). Common in coral-reef areas of the Ryukyu Archipelago of Japan, the southern coast, and eastern islands (Lyudao and Lanyu) of Taiwan, but few in other areas. Specimens collected from 0–20 m depth.

#### Remarks.

**Figure 3.**
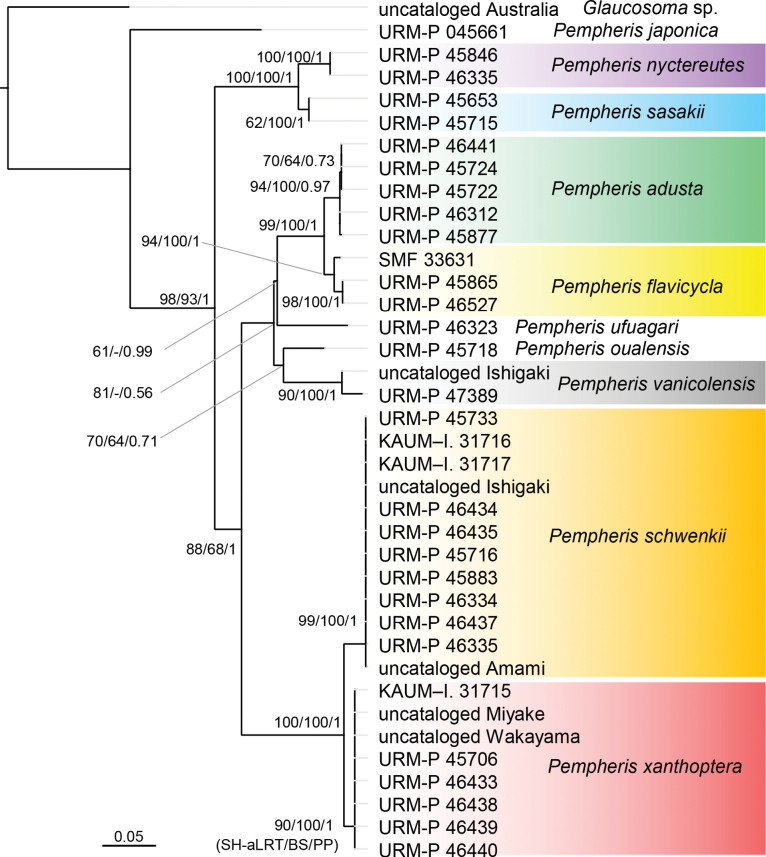
The Maximum-Likelihood (ML) tree of *Pempheris* species recovered from mitochondria *16S* and *COI*. Values of the Shimodaira–Hasegawa-like approximate likelihood ratio test (SH-aLRT) for the ML tree, bootstrap values (BS) of the NJ tree, and the posterior probability (PP) for the BI tree are indicated at the nodes unless the branch lengths are < 0.01. The museum voucher number of specimens are listed next to the taxon name.

Although the taxonomic position of *P.adusta* was unsettled for a long time, the holotype (RMNH.PISC.6161: Ambon, Indonesia) matches well with the specimens in Koeda et al. (2013b). The original description of the species is also supported as follows: figures of six species (*P.mangula* Cuvier, 1829, *P.schwenkii*, *P.vanicolensis*, *P.adusta*, *P.otaitensis* Cuvier, 1831, and *P.oualensis*) were illustrated in the plate of Bleeker (1877); the first three species have no black blotch and the latter three species possess a black blotch on the pectoral-fin base. This was also mentioned in the text descriptions, where he specified that *P.oualensis* and *P.otaitensis* had a black blotch, but *P.adusta* has a black or brown blotch on the pectoral-fin base visible in the figure and clearly corresponding with the descriptions of the three species. Koeda et al. (2014) indicated that *Pempherisadusta* was widely distributed species from the Indian to Pacific oceans and had intraspecific variations in their morphology. The Pacific group differs from the Indian Ocean group in the following characters and individuals from Andaman Sea have characters intermediate between these two groups: pored lateral-line scales 51–62 (vs 56–63 in Indian Ocean; 53–57 in Andaman Sea); scale rows above lateral line usually 4½ (vs usually 5½ in Indian Ocean; 4½ in Andaman Sea); usually no blackish band on anal fin (vs distinct blackish band on outer margin of anal fin in Indian Ocean and Andaman Sea); blackish band on anal-fin base (no band on anal-fin base in Indian Ocean and Andaman Sea); and anterior margin of dorsal fin blackish (tip of dorsal fin blackish in Indian Ocean and Andaman Sea). Furthermore, Koeda et al. (2014) demonstrated that nucleotide sequences of specimens collected from the Red Sea, the Andaman Sea, and the Pacific Ocean showed only 0.4% difference in mitochondrial *16S* ribosomal DNA. Coupled with the observation of small morphological differences, they considered *Pempherisflavicycla* Randall, Satapoomin & Alpermann, 2014 (type locality: Mafia Island, Chole Islands, Chole Bay, Tanzania) to be a junior synonym of *P.adusta*. However, Randall et al. (2014) countered this opinion based on the 2.5% difference in *COI* sequences, stating that *P.adusta* is a species in Pacific Ocean, *P.flavicycla* is the valid species in Indian Ocean, and described the Andaman group as a subspecies *Pempherisflavicyclamarisrubri* Randall, Bogorodsky & Alpermann, 2014. Based on the genetic comparison incorporating the *COI* and *16S* genes in the present study, it was shown that there is more than a 2% genetic difference between *P.adusta* and *P.flavicycla* (Fig. 3), which is not a subtle difference when compared to differences among other similar species in the genus. Although the issue of overlapped morphological differences remains in *P.adusta* and *P.flavicycla*, it is reasonable to support the opinion of Randall et al. (2014) that *P.flavicycla* is considered as a valid species distributed in Indian Ocean at the present time.

### ﻿ Pempheris familia

Taxon classificationAnimaliaPerciformesPempheridae

Koeda & Motomura, 2017

DEE4D131-082B-5440-B017-D10D9D9A8FF6

**Figure 4.**
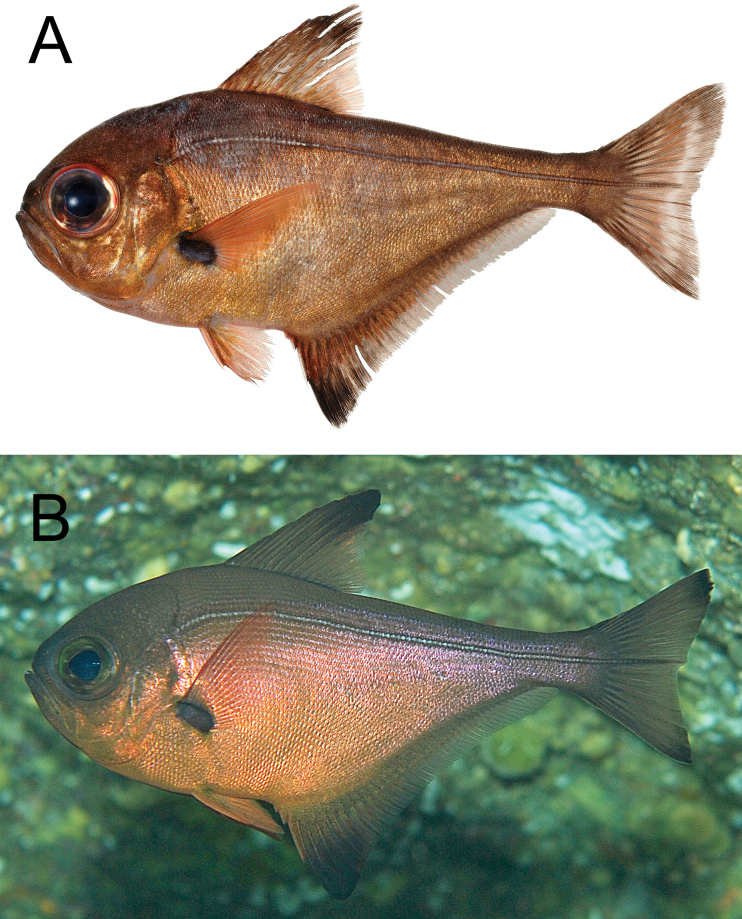
*Pempherisfamilia***A** fresh specimen (KAUM–I. 74713, 153.1 mm SL, holotype, Ototo-jima Island, Ogasawara Islands, Japan, photo taken by K. Kuriiwa) and **B** underwater photograph (Ani-jima Island, Ogasawara Islands, Japan).

**Figure 5.**
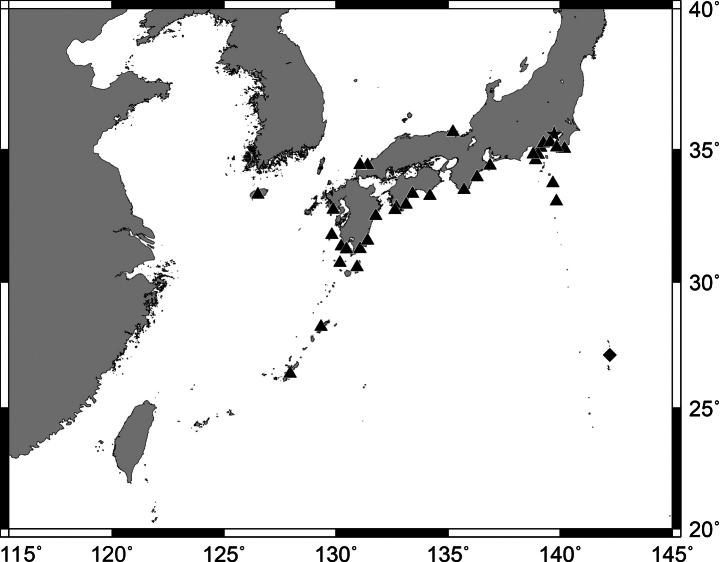
Distribution of *Pempherisfamilia* (diamond) and *Pempherisjaponica* (triangles and star for type locality) based on the collection locality of the specimens.

Figs 4 , 5 , Suppl. material 2 Standard Japanese name: Bonin-hatampo

Pempheris familia Koeda & Motomura, 2017a: figs 1–3 (type locality: off Ototo-jima Island, Ogasawara Islands, Japan); Koeda 2018b: 299, unnumbered fig.
Pempheris japonica (not Döderlein, 1883): Toyama 1937: 36; Kurata et al. 1971: 25; Zama and Fujita 1977: 102; Randall et al. 1997: 35; Hatooka and Yagishita 2013: 983 (in part); Koeda 2017a: 6, fig. 1 (lower fig.).

#### Diagnosis.

Counts of holotype and paratype are given in Table 1 of Koeda and Motomura (2017a). Dorsal-fin rays VI, 9–10; anal-fin rays III, 35–36; pectoral-fin rays 17; pored lateral-line scales 84–88; scale rows above lateral line 14–15; scale rows below lateral line 28–30; predorsal scales 50–55; circumpeduncular scales 26; gill rakers 12–13+22–26 = 34–39; head length 28.8–30.1% SL; body depth 42.9–43.7% SL; eye diameter 46.8–47.6% HL; upper jaw length 50.0–56.3% HL; maximum 153 mm SL; scales strongly ctenoid, adherent, divided into basal and distal halves (Koeda et al. 2013a: fig. 2b); body copper; distinct blackish blotch on pectoral-fin base; tip of dorsal and anal fins broadly black, and rest brown; narrow band of villiform teeth in jaws; abdomen cross-sectional outline U-shaped.

#### Distribution.

Endemic to the Ogasawara Islands (Fig. 5).

#### Remarks.

*Pempherisfamilia* is a second species of the species group that is characterized by strongly ctenoid, adherent body scales with distinct basal and distal portions (see Koeda et al. 2013a: fig. 2b) in the Northern Hemisphere shared only with *P.japonica* (Koeda et al. 2012a, 2013a; Koeda and Motomura 2017a). The remaining six species of this group are endemic to Australia, New Zealand, or French Polynesia (Mooi and Jubb 1996; Mooi 1998, 2000). Although *P.familia* shares most morphological characteristics with *P.japonica*, the former can be clearly distinguished from the latter in scale counts and the distinct black blotch on pectoral-fin base (Koeda and Motomura 2017a).

### ﻿ Pempheris japonica

Taxon classificationAnimaliaPerciformesPempheridae

Döderlein, 1883

FE95D3FF-CBEE-5A1D-84B4-E87033D6FB17

**Figure 6.**
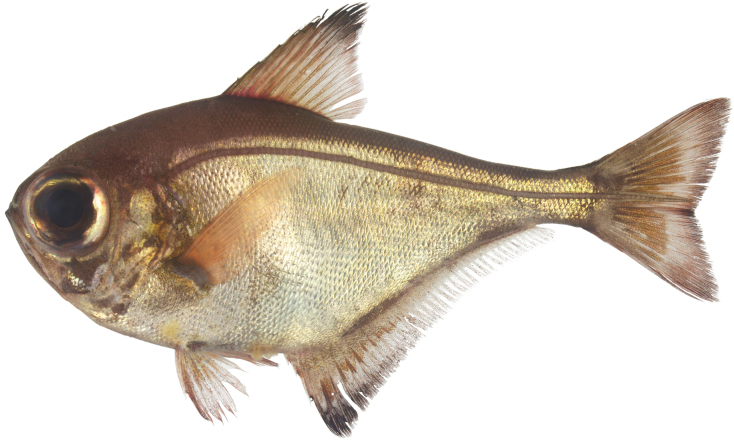
Fresh specimen of *Pempherisjaponica*, KAUM–I. 89834, 125.0 mm SL, Nakakoshiki-jima Island, Koshiki Islands, Japan.

Figs 5 , 6 , Suppl. material 2 Standard Japanese name: Tsumaguro-hatampo

Pempheris japonica Döderlein, 1883: 125 (type locality: Tokyo, Japan); Jordan et al. 1913: 137; Tanaka 1931: 25; Uchida 1933: 208; Okada 1938: 179; Okada and Matsubara 1938: 179; Matsubara 1955: 590; Tominaga 1963: 278, fig. 6; Takemura and Yasuda 1965: 159; Masuda et al. 1975: 199, pl. 33-B; Hayashi 1984 (in part): 160, pl. 151-C; Kohno 1986: 135, fig. 1; Masuda and Kobayashi 1994: 180, fig. 4; Mochizuki 1995: 389, unnumbered fig.; Hatooka 1997: 380, unnumbered fig.; Hatooka 2002 (in part): 877; Senou et al. 2002: 212; Senou et al. 2006b: 463; Aramata 2007: 171, unnumbered fig.; Yoshino 2008: 211, unnumbered fig.; Koeda et al. 2010a: 74; Koeda et al. 2010b: 81; Motomura et al. 2010: 131; Takagi et al. 2010: 69, unnumbered figs; Kohno et al. 2011: 208, unnumbered fig.; Koeda et al. 2012a: 65; Hatooka and Yagishita 2013 (in part): 983; Koeda et al. 2013b: 235; Motomura et al. 2013: 168, unnumbered fig.; Kawano et al. 2014: 48; Koeda et al. 2014: 327; Koeda and Motomura 2015: 139; Koeda et al. 2015: 275; Ikeda and Nakabo 2015: 159, figs 5–7; Takeuchi et al. 2015: 8; Koeda 2017b: 190, unnumbered fig.; Koeda and Motomura 2017a; Fujiwara et al. 2018: 58, Fig. 8L; Kagoshima City Aquarium Foundation 2018: 210, unnumbered fig.; Koeda 2018b: 298, unnumbered fig.; Nakae et al. 2018: 266; Koeda 2018c: 341, unnumbered figs; Murase et al. 2019: 132, fig. 282; Koeda 2020a: 408, unnumbered figs; Sonoyama et al. 2020: 78; Murase et al. 2021: 166, fig. 338; Koeda 2022: 158, unnumbered fig.; Koeda et al. 2022: 6; Motomura 2023: 127; Sakurai et al. 2024: 77, fig. 4D.
Catalufa umbra Snyder, 1911: 528 (Misaki, Japan).
Catalufa japonica (not Döderlein, 1883): Jordan and Hubbs 1925: 227.
Pempheris umbra (not Snyder, 1911): Okada 1938: 179; Okada and Matsubara 1938: 179; Matsubara 1955: 59, pl. 54 (fig. 189); Takemura and Yasuda 1965: 159; Abekawa and Nishi 1969: 24.

#### Diagnosis.

Counts of syntypes and non-types are given in Table 1 of Koeda and Motomura (2017a). Dorsal-fin rays VI, 10–12; anal-fin rays III, 34–40; pectoral-fin rays 16–17; pored lateral-line scales 69–82; scale rows above lateral line 12–13; scale rows below lateral line 26–30; predorsal scales 40–44; circumpeduncular scales 22–24; gill rakers 8–12+19–25 = 28–35; head length 28.3–31.4% SL; body depth 43.2–47.7% SL; eye diameter 38.1–50.0% HL; upper jaw length 50.0–56.3% HL; maximum 153 mm SL; scales strongly ctenoid, adherent, divided into basal and distal halves (see Koeda et al. 2013a: fig. 2b); body copper; no or faint blackish blotch on pectoral-fin base; tip of dorsal and anal fins broadly black, remainder brown; narrow band of villiform teeth in jaws; abdomen cross-sectional outline U-shaped.

#### Distribution.

Endemic to the region from southern Korea to southern Japan. In Japanese waters, *P.japonica* is distributed in the Pacific coast (north to Ishinomaki in Miyagi Prefecture, south to Kagoshima Prefecture), Japan Sea coast (east to Miyazu in Kyoto Prefecture, west to Tsuno-shima Island in Yamaguchi Prefecture), Tsushima Island, East China Sea coast (north to Nagasaki, south to Kagoshima prefectures), Miyake-jima and Hachijo-jima islands in Izu Islands, Tanega-shima, Yaku-shima, Iou-jima, Amami-oshima, and Okinawa-jima in Ryukyu Archipelago (very rare in the latter two islands) (Fig. 5).

#### Remarks.

Döderlein (1883) described *P.japonica* based on syntypes collected from Tokyo Bay, and Snyder (1911) described *Catalufaumbra* based on the holotype collected from Kanagawa Prefecture. The type specimens of the two nominal species were compared in the present study, and no differences were observed. Therefore, *C.umbra* is confirmed as a junior synonym of *P.japonica*, in agreement with Tominaga (1963).

Snyder (1912) and Shao et al. (2008) reported *P.japonica* in the fish checklists of Okinawa Island and southern Taiwan, respectively. However, the specimens they observed and identified as *P.japonica* (CAS-SU 22002; ASIZP 61383) are identified as *P.schwenkii* and *P.vanicolensis*, respectively (Koeda et al. 2012a). Subsequently, Randall et al. (1997) reported *P.japonica* from the Ogasawara Islands. However, that report was not based on specimens and/or underwater observations (refer to pers. comm. of R. Mooi on p. 35) and was probably the misidentification of *P.familia* which is similar to *P.japonica*. Although Hayashi (1984) and Hatooka (2000, 2002) included the Philippines, and Hatooka and Yagishita (2013) included Taiwan in the distributional range of *P.japonica*, specimens of *P.japonica* from these localities have never been discovered. Our results indicate that *P.japonica* is not distributed in these localities, and the species is endemic to Japan and southern Korea. Compared to the distribution of other species of the same genus found in the Northern Hemisphere, this species can be said to be the most temperate species adapted to the lowest water temperatures. Until the 2010s, this species was distributed only as far as the Boso Peninsula, a trend known for many tropical fish species. However, in recent years, new distribution records have been reported from Fukushima and Miyagi Prefectures, likely a result of northward range expansion due to global warming.

### ﻿ Pempheris nyctereutes

Taxon classificationAnimaliaPerciformesPempheridae

Jordan & Evermann, 1903

942D4C72-5262-5279-B581-CFFDC4743B84

**Figure 7.**
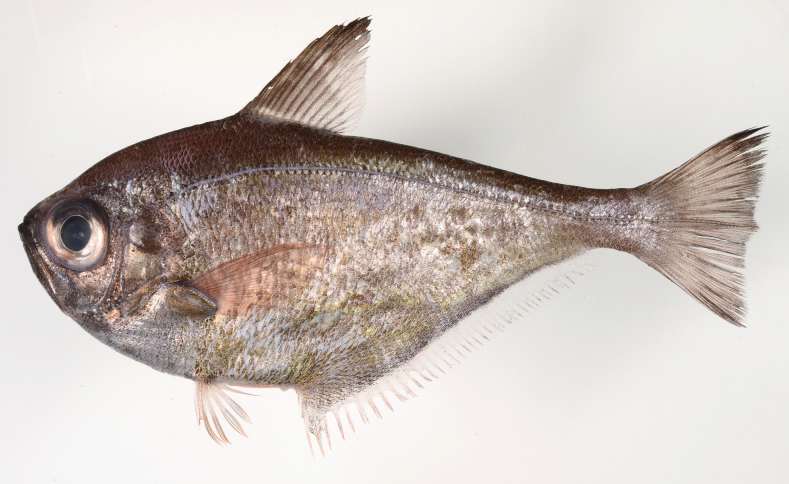
Fresh specimen of *Pempherisnyctereutes*, NMMB-P 27469, 149.4 mm SL, Penghu, Taiwan.

**Figure 8.**
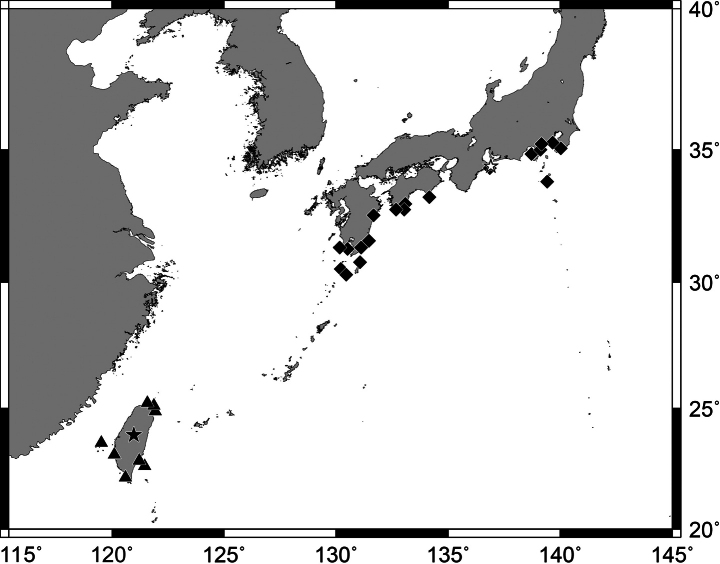
Distribution of *Pempherisnyctereutes* (solid triangles and solid star for holotype locality) and *Pempherissasakii* (solid diamonds and open star for holotype locality) based on the collection localities of the specimens.

Figs 7 , 8 ; Table 1 , Suppl. material 2 Standard Japanese name: Taiwan-hatampo

Pempheris nyctereutes Jordan & Evermann, 1903: 339, fig. 14 (type locality: Taipei City [Hokoto], Taiwan); Okada 1938: 179; Okada and Matsubara 1938: 179; Matsubara 1955:590; The Marine Ecological Researching Society of Kagoshima University 1966: 19; Shen 1993: 390, unnumbered fig.; Koeda et al. 2013a: 237; Koeda et al. 2014: 327; Tominaga 1963: 281, fig. 8; Hayashi 1984 (in part): 161; Randall and Lim 2000: 622; Hatooka 2002 (in part): 877; Hatooka and Yagishita 2013 (in part): 984; Shen and Wu 2011: 497, unnumbered fig.; Chiang et al. 2014: 183, unnumbered fig.; Koeda 2019: 927, unnumbered figs; Koeda 2020b: 927, unnumbered figs; Koeda et al. 2022: 9.
Pempheris schwenkii (not Bleeker, 1855): Lee 1996: 97, unnumbered fig.

#### Diagnosis.

Counts of holotype and non-types are given in Table 1. Dorsal-fin rays VI, 9; anal-fin rays III, 42–44; pectoral-fin rays 18–20; pored lateral-line scales 72–81 usually > 79; scale rows above lateral line 8 1/2–9 1/2; scale rows below lateral line 23–28; circumpeduncular scales 22–24; gill rakers 8+19–20 = 27–28; head length 30.0–30.9%; body depth 44.6–46.9%; eye diameter 41.0–46.4%; upper jaw length 51.3–53.5%; maximum 161 mm SL; snout sharp; scales weakly ctenoid, deciduous, thin, semicircular in shape, far wider than long (see Koeda et al. 2013a: fig. 2a); body silver to dark brown in fresh specimens; tip and anterior margin of dorsal fin zonally blackish; anal fin pale with faint black band on base; paired fins pink; posterior half of caudal fin dusky; body light brown in fixed specimen; black pigmentation on each fin usually persistent, but not in holotype; no blackish blotch on pectoral-fin base; narrow band of villiform teeth in jaws; abdomen cross-sectional outline is V-shaped.

#### Distribution.

Recorded only from Taiwan, Hong Kong, and Vietnam. In Taiwanese waters, this species is known from Nang-fang-ao in Yilan County, Keelung City, Aodi, Wanli, and Gongliao in New Taipei City, Tainan County, Fugang in Taitung County, Hengchung and Maobitou in Pingtung County, Lyudao, and Penghu (Fig. 8).

#### Remarks.

The taxonomic status of *P.nyctereutes* and *P.sasakii* that have similar morphology have been commonly confused. A comparison between these two species is discussed in the remarks for *P.sasakii*. *Pempherisnyctereutes* is sometimes collected by fisherman in southern Taiwan as bycatch with *P.schwenkii* and *P.adusta*, which are mainly distributed in coral-reef areas, suggesting that *P.nyctereutes* might be distributed in coral-reef areas. This species is also known from Ha Long Bay in Vietnam (FRLM 49700; Koeda 2018d) and Hong Kong (BMNH 1939.3.23.48). Okada (1938), Okada and Matsubara (1938), and Matsubara (1955) admitted *P.nyctereutes* and *P.sasakii* as valid species being endemic to Taiwan and Japan, respectively, and Okada (1938) gave the Japanese name “Taiwan-hatampo” and “Mie-hatampo” to them. Tominaga (1963) later proposed “Takasago-hatampo” for *P.nyctereutes* as a new Japanese name without any reasons (probably overlooking the original designation), and Koeda et al. (2022) followed that. Based on Rule 6 of the guidelines for the naming of standard Japanese names for fishes (The Ichthyological Society of Japan 2021), “Taiwan-hatampo” proposed by Okada (1938) should be adopted for *P.nyctereutes*.

### ﻿ Pempheris oualensis

Taxon classificationAnimaliaPerciformesPempheridae

Cuvier, 1831

3AA554BF-A056-542B-B1F3-B865EB498645

**Figure 9.**
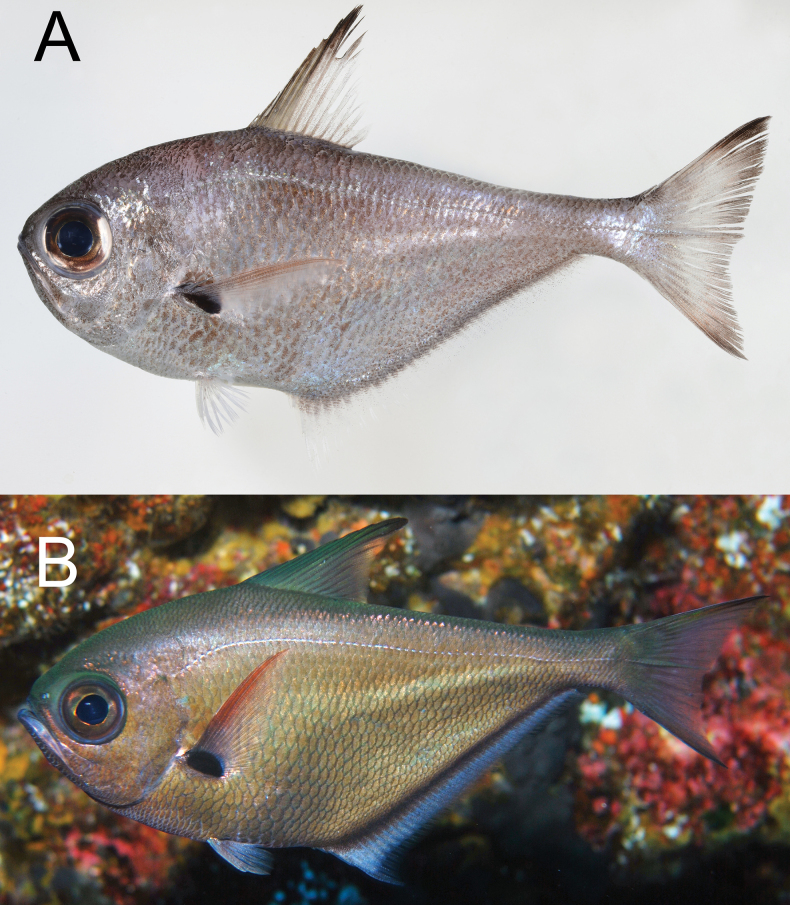
*Pempherisoualensis***A** fresh specimen (NMMB-P 27821, 175.4 mm SL, Hengchung, Pingtung, Taiwan) and **B** underwater photograph (Dobuiso, Ogasawara Islands, Japan).

**Figure 10.**
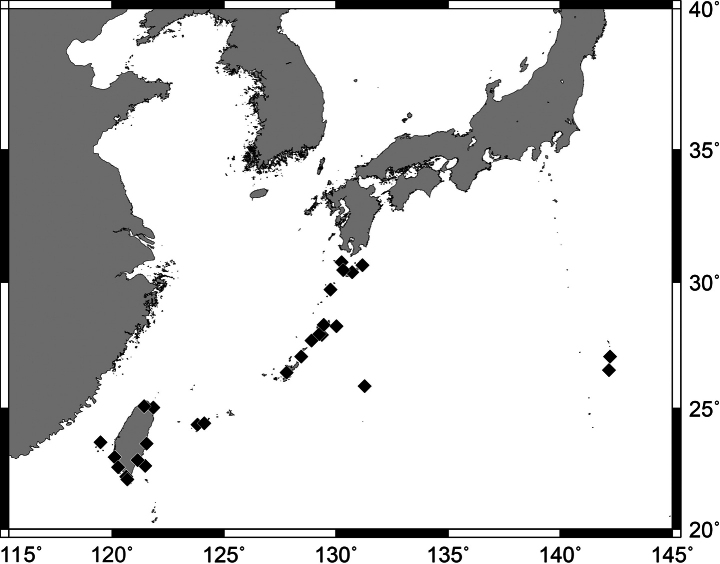
Distribution of *Pempherisoualensis* based on the collection locality of the specimens.

Figs 9 , 10 , Suppl. material 2 Standard Japanese name: Yume-hatampo

Pempheris oualensis Cuvier in Cuvier & Valenciennes, 1831: 299 (type locality: Kosrae, Caroline Islands); Masuda et al 1975 (in part): 199; Hayashi 1984 (in part): 161; Chen et al. 1995 (probably in part): 25; Shao et al. 2008: 254; Koeda et al. 2010a: 72, fig. 1; Koeda et al. 2010b: 81; Koeda et al. 2012a: 71; Koeda et al. 2013a: 231, fig. 1b; Koeda et al. 2013b: 126; Koeda et al. 2014: 327; Koeda and Motomura 2015: 139; Koeda et al. 2015: 276, fig. 1; Koeda et al. 2016b: 50, fig. 225; Koeda and Motomura 2017a; Koeda 2017a: 7, fig. 2 (upper fig.); Kimura et al. 2017: 119, fig. 7; Planning and Tourism Division of Kikai Town 2017: 4, unnumbered figs; Nakae et al. 2018: 266; Koeda 2018a: 194, unnumbered figs; Koeda 2018b: 298, unnumbered fig.; Mochida and Motomura 2018: 30; Koeda 2019: 928, unnumbered figs; Motomura and Uehara 2020: 45; Koeda 2020b: 928, unnumbered figs; Motomura 2023: 129.
Pempheris otaitensis (not Cuvier, 1831): Schmidt 1913: 121; Randall et al. 1997 (in part): 35, pl. 8 (fig. F).
Pempheris sp.: Hatooka 2002 (in part), 878; Motomura et al. 2010 (in part): 131, fig. 253; Hatooka and Yagishita 2013 (in part): 984.

#### Diagnosis.

Counts of holotype and non-types are given in Table 2 of Koeda et al. (2010a). Dorsal-fin rays VI, 9–10; anal-fin rays III, 38–46; pectoral-fin rays 17–19; pored lateral-line scales 60–72; scale rows above lateral line 5 1/2–7 1/2, usually 6 1/2; scale rows below lateral line 13–17; predorsal scales 33–44; circumpeduncular scales 18–22; gill rakers 8–9+20–22 = 28–31; head length 27.7–31.5%; body depth 40.9–48.2%; eye diameter 36.4–43.9%; upper jaw length 50.0–56.0%; maximum 208 mm SL, usually < 180 mm SL; scales weakly ctenoid, deciduous, thin, semicircular in shape, far wider than long (Koeda et al. 2013a: fig. 2a); body silver; distinct blackish blotch on pectoral-fin base; anterior margin of dorsal fin zonally blackish; blackish band on anal-fin base with rarely blackish band on its margin; upper margin of pectoral fin dusky; villiform tooth band extending outside lips on large specimen; abdomen cross-sectional outline V-shaped.

**Table 2.** Counts of *Pempherisschwenkii* and *P.xanthoptera*.

	*P.schwenkii*		*P.xanthoptera*	
	Syntypes	Non-types	Holotype	Non-types
Number of individuals	2	232	1	355
Number of individuals	87.2, 89.7	20.4–125.9	116.8	28.2–136.6
Standard length				
Dorsal fin rays	VI, 9	VI–VII, 9–10	VI, 9	VI–VII, 9
Anal fin rays	III, 35–36	III, 35–42	III, 38	III, 35–42
Pectoral fin rays	17–18	16–18	18	16–19
Left pored lateral-line scales	48	44–53	48	45–54
Right pored lateral-line scales	48–49	45–53	47	46–54
Scale above lateral line	3 1/2	3 1/2–4 1/2	3 1/2	3 1/2
Scale rows below lateral line	10	10–13	10	10–13
Circumpeduncular scales	12	10–12	12	12–14
Gill rakers	7+18–19	6–9+18–22	N/A	7–9+18–21

#### Distribution.

Widely distributed in the western to central Pacific Ocean (not in the Hawaiian Islands), and Christmas Island and Cocos-Keeling Island in the Indian Ocean. In Japanese waters, this species is known from Tanega-shima to Yonaguni-jima islands in the Ryukyu Archipelago, Minamidaito-jima Island in the Daito Islands, and Haha-shima and Chichi-jima islands in the Ogasawara Islands. In Taiwanese waters, this species is known from Nang-fang-ao in Yilan County, Yeh Liu in New Taipei City, Tainan County, Chung-chou in Kaohsung County, Hengchung, and Kenting in Pingtung County, Fugang in Taitung County, and Lanyu (Fig. 10).

#### Remarks.

This species has similar characters as *Pempherisotaitensis* Cuvier, 1831 and *Pempherisufuagari* which share a distinct black blotch on pectoral-fin base and whose large body sizes reaches > 160 mm SL. However, *P.oualensis* is unique in having a dark coloration on the upper margin of its pectoral fin and a villiform tooth band extends outside the lips on large specimens. The scale count of this species varies among populations that specimens collected from the northwestern Pacific have 61–66 pored lateral-line scales, but specimens collected from southern Pacific have 67–71. Additionally, specimens collected from Andaman Sea have 5½ scale rows above lateral line, whereas those from the Pacific Ocean have 6½ or 7½, with the frequency of specimens with 7½ scale rows above lateral line being lower in the northwestern Pacific than in southern areas. In particular, the Andaman population may be a species distinct from the Pacific populations, but more specimens and genetic evidence are necessary to discuss whether the differences are interspecific or intraspecific.

Although Okada (1938) gave the Japanese name “Ryukyu-hatampo” to *P.oualensis* which was listed in Snyder (1912), specimen (USNM 75468) used for the list was re-identified to *P.adusta* (Koeda et al. 2013b). Therefore, the standard Japanese name “Ryukyu-hatampo” was adopted for *P.adusta* and the standard Japanese name “Yume-hatampo” was provided to *P.oualensis* by Koeda et al. (2010a) who first reported this species from Japanese waters. This species is the largest species of *Pempheris* that reaches > 200 mm SL; the largest specimen was collected from the Ogasawara Islands and measured 208.8 mm SL (KAUM–I. 74584).

### ﻿ Pempheris sasakii

Taxon classificationAnimaliaPerciformesPempheridae

Jordan & Hubbs, 1925

F34475CC-5F34-5A94-84F9-95135C7D54F4

**Figure 11.**
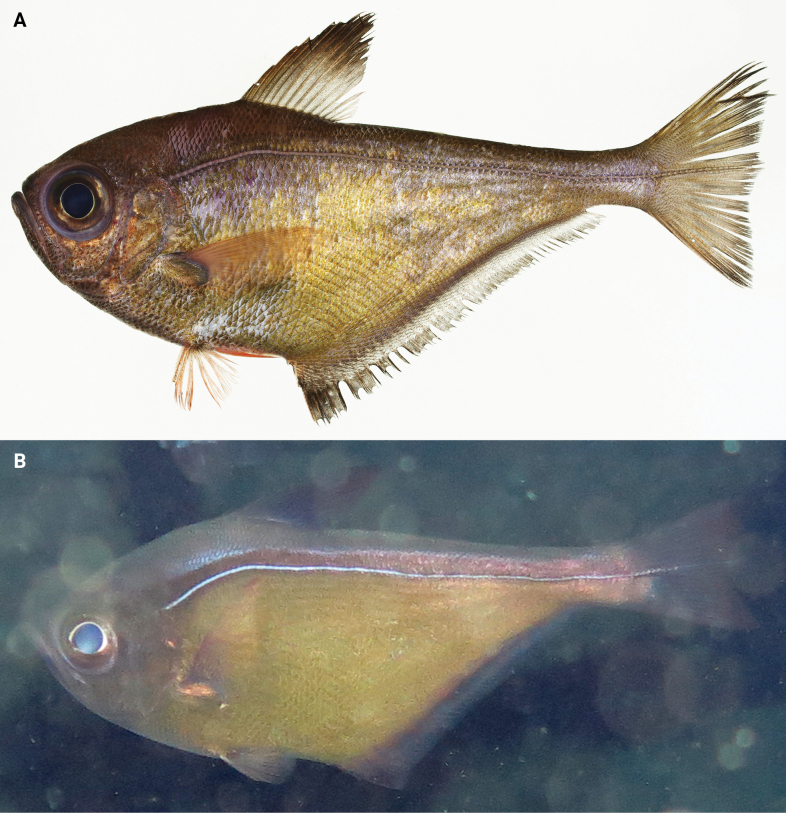
*Pempherissasakii***A** fresh specimen (KAUM–I. 94368, 126.7 mm SL, Uchinoura Bay, Kimotsuki, Kagoshima) and **B** underwater photograph (lower: Minamisatsuma, Kagoshima, Japan).

Figs 8 , 11 , Suppl. material 2 Standard Japanese name: Mie-hatampo

Liopempheris sasakii Jordan & Hubbs, 1925: 228, pl. 10, fig. 1 (type locality: Toba, Mie Prefecture, Japan); Tanaka 1931: 25.
Pempheris sasakii : Uchida 1933: 217; Okada 1938: 179; Okada and Matsubara 1938: 180; Matsubara 1955: 590; Tominaga 1963: 283, fig. 10; Koeda et al. 2013a: 231; Koeda et al. 2014: 327.
Pempheris nyctereutes (not Jordan & Evermann, 1903): Hayashi 1984 (in part): 160, pl. 350-G; Hatooka 2002 (in part): 879; Senou et al. 2006b: 463; Koeda et al. 2010a: 75; Koeda et al. 2010b: 81; Hatooka and Yagishita 2013 (in part): 984; Koeda and Motomura 2015: 139; Koeda et al. 2015: 275; Ikeda and Nakabo 2015: 160, figs 4–6; Kaburagi 2016: 99, upper fig. (without scientific name; shown as “Mie-hatampo”); Kimura et al. 2017: 119, fig. 6; Kagoshima City Aquarium Foundation 2018: 210, unnumbered fig.; Koeda 2018b: 298, unnumbered fig.; Koeda 2018c: 342, unnumbered figs; Murase et al. 2019: 132, fig. 285; Koeda 2020a: 409, unnumbered figs; Murase et al. 2021: 166, fig. 341; Koeda 2022: 158, unnumbered fig.; Koeda et al. 2022: 9; Motomura 2023: 128.

#### Diagnosis.

Counts of holotype and non-types are given in Table 1. Dorsal-fin rays VI–VII, 9–10; anal-fin rays III, 40–46; pectoral-fin rays 17–20; pored lateral-line scales 67–78, usually fewer than 73; scale rows above lateral line 8½–10½; scale rows below lateral line 19–22; circumpeduncular scales 24; gill rakers 7–9+19–22 = 28–30; head length 28.1–29.7%; body depth 40.6–44.6%; eye diameter 38.9–43.2%; upper jaw length 50.0–55.6%; maximum 170 mm SL; snout sharp; scales weakly ctenoid, deciduous, thin, semicircular in shape, far wider than long (see Koeda et al. 2013a: fig. 2a); dorsal half of body brown, with golden reflection in fresh specimen; ventral half golden; tip and anterior margin of dorsal fin zonally blackish; faint blackish band on anal-fin base; dusky band on outer edge of anal fin; paired fins pink; posterior half of caudal fin dusky; body pale brown in fixed specimen; black pigmentation on each fins usually persistent, but not in holotype; no blackish blotch on pectoral-fin base; narrow band of villiform teeth in jaws; abdomen cross-sectional outline V-shaped.

#### Distribution.

Endemic to southern Japan known from Tateyama at Boso Peninsular in Chiba Prefecture, Misaki and Manazuru in Kanagawa Prefecture, Nishi-izu at Izu Peninsular in Shizuoka Prefecture, Toba and Shima in Mie Prefecture, Muroto, Susaki, Tosashimizu, Iburi, and Otsuki in Kochi Prefecture, Nobeoka and Nango in Miyazaki Prefecture, Uchinoura Bay, Kagoshima Bay, Ibusuki, and Minami-satsuma in Kagoshima Prefecture, Miyake-jima Island in Izu Islands, Tanega-shima, Yaku-shima, and Kuchinoerabu-jima islands in the northern Ryukyu Archipelago (Fig. 8).

#### Remarks.

*Pempherissasakii* has been commonly confused with *P.nyctereutes* and has been presumed to be the junior synonym (e.g., Hayashi 1984), even though both species were described by the same first author. Only Tominaga (1963) showed the difference in anal fin coloration (*P.sasakii*: margin of anal fin fuscous vs *P.nyctereutes*: margin of anal fin pale) between these two species and described the details of both as valid species. In our morphological observations, however, several *P.nyctereutes* specimens have dusky margins on the anal fin; thus, this character was not diagnostic for identifying those species. The present comparison based on both species indicated that these two species can be distinguished by the counts of scale rows below lateral line (19–22 in *P.sasakii* vs 25–27 in *P.nyctereutes*) with modal difference of pored lateral-line scales (72–81 usually > 79 vs 67–78 usually fewer than 73). The coloration of the species slightly differs in that the former has golden body (sometimes silverish) in fresh condition, but the latter has copper to silver coloration: compare Figs 7, 11). Although the morphological differences between the two species are very few, the molecular analyses strongly supported the intraspecific difference which revealed that sequences of *P.nyctereutes* and *P.sasakii* differed by more than 3.1% over mitochondrial *16S* ribosomal DNA and *COI*, comprising different monophyletic groups (Fig. 3).

*Pempherissasakii* is widely distributed in the Pacific coast of southern Japan, and commonly collected by set nets (but not abundant compared to *P.xanthoptera*). However, no specimens of this species have ever been collected from the Japan Sea coast, East China Sea coast, and the Ryukyu Archipelago. The distributions of both *P.sasakii* and *P.nyctereutes* are clearly isolated from each other (Fig. 8).

### ﻿ Pempheris schwenkii

Taxon classificationAnimaliaPerciformesPempheridae

Bleeker, 1855

F87C3014-64B2-5BC9-9D84-39495B1E94C6

**Figure 12.**
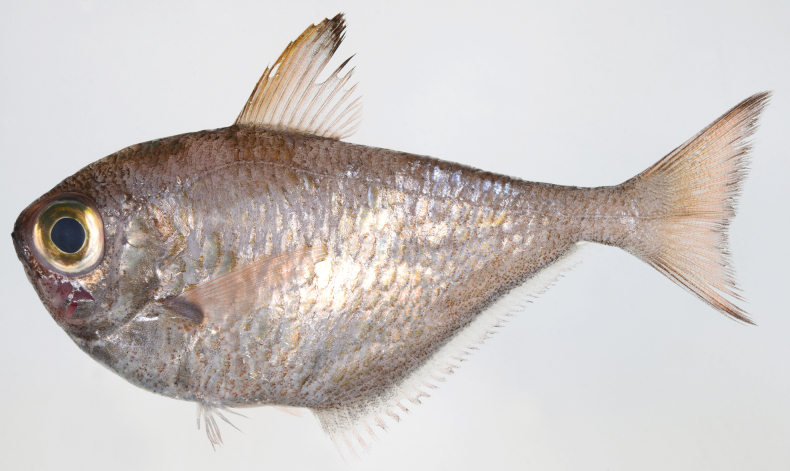
Fresh specimen of *Pempherisschwenkii*, NMMB-P 27013, 100.3 mm SL, Hengchung, Pingtung, Taiwan.

**Figure 13.**
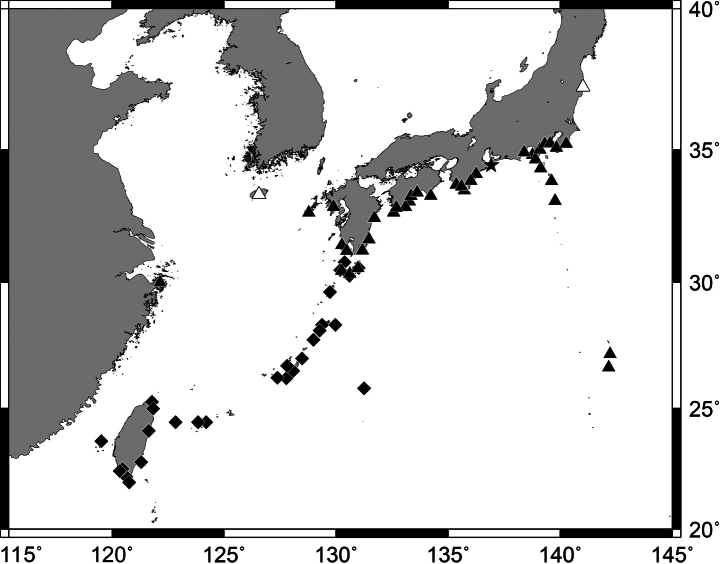
Distribution of *Pempherisschwenkii* (diamonds) and *P.xanthoptera* (solid triangles and star for type locality) based on the collection locality of the specimens. Open triangles for literature records of *P.xanthoptera*.

Figs 12 , 13 , Suppl. material 2 Standard Japanese name: Minami-hatampo

Pempheris schwenkii Bleeker, 1855: 314 (type locality: Batu Islands, Sumatera Utara Province, Indonesia); Hatooka 1997 (in part): 380, unnumbered fig. (p. 381, lower middle fig.); Randall and Lim 2000: 622; Hatooka 2002 (in part): 878; Yoshigou and Nakamura 2002: 107; Yoshigou and Nakamura 2003: 49; Yoshigou 2004: 19; Chen 2003: 134, unnumbered fig.; Senou et al. 2006a: 77; Senou et al. 2007: 56; Shao et al. 2008: 254; Yoshino 2008 (in part): 211; Ito 2009: 80, unnumbered fig.; Chen et al. 2010: 265, fig. C, E; Koeda et al. 2010a: 75; Koeda et al. 2010b: 81; Motomura et al. 2010: 131, fig. 252; Shen and Wu 2011: 498, unnumbered fig.; Koeda et al. 2012a: 71; Koeda et al. 2012b: 1086; Miura 2012: 59 (without scientific name; shown as “Minami-hatampo”); Chiang et al. 2014: 183, unnumbered fig.; Hatooka and Yagishita 2013 (in part): 984; Koeda et al. 2013a: 235; Koeda et al. 2013b: 222, fig. 1; Koeda et al. 2013c: 126; Motomura et al. 2013 (in part): 168; Shao et al. 2013 (in part): 163, unnumbered fig. (lower); Koeda et al. 2014: 314; Koeda and Motomura 2015: 139 ; Motomura and Matsuura 2014: 271, unnumbered figs; Koeda et al. 2015: 275; Koeda et al. 2016a: 519; Koeda et al. 2016c: 8, fig. 3H; Koeda and Motomura 2017a; Koeda and Motomura 2017b: 266, fig. 3D, E; Kimura et al. 2017 (in part): 120, fig. 1; Planning and Tourism Division of Kikai Town 2017: 4, unnumbered figs; Nakae et al. 2018: 266; Koeda 2018b: 298, unnumbered fig. (lower right fig.); Koeda 2018b: 194, unnumbered figs; Mochida and Motomura 2018: 30; Koeda 2019: 929, unnumbered figs; Fujiwara and Motomura 2020: 28; Koeda 2020b: 929, unnumbered figs; Motomura and Uehara 2020: 45; Koeda et al. 2022: 10; Motomura 2023: 129 (in part).
Pempheris adusta (not Bleeker, 1877): Shimose 2021: 122, fig. B.
Pempheris japonicus (not Döderlein, 1883): Snyder 1912: 497.
Pempheris oualensis (not Cuvier, 1831): Chen 2003: 134, unnumbered fig.; Shao and Chen 2003: 255, unnumbered figs; Yang et al. 2013: 167, unnumbered fig.
Liopempheris vanicolensis (not Cuvier, 1831): Jordan and Hubbs 1925: 229.
Pempheris vanicolensis (not Cuvier, 1831): Okada 1938: 179; Okada and Matsubara 1938: 179 (in part); Matsubara 1955: 590 (in part); Aoyagi 1948: 49; The Marine Ecological Researching Society of Kagoshima University 1966: 19; The Marine Ecological Researching Society of Kagoshima University 1967: 32; Takahashi 1970: 58; Chen et al. 2010: 266, fig. A; Chang et al 2011: 46.
Pempheris xanthoptera Tominaga, 1963 (in part paratypes): 287; Masuda et al 1975 (in part): 199, pl. 33-C; Yoshino et al. 1975: 75; Hayashi 1984 (in part): 160.
Pempheris sp.: Uchida 1933: 218 (in part).

#### Diagnosis.

Counts of holotype and paratypes are given in Table 2. Dorsal-fin rays VI–VII, very rarely VII, 9–10, very rarely 10; anal-fin rays III, 35–42, usually > 37; pectoral-fin rays 16–18; pored lateral-line scales 44–53; scale rows above lateral line 3½ or 4½, very rarely 4½; scale rows below lateral line 10–13; predorsal scales 23–30; circumpeduncular scales 10–12; gill rakers 6–9+18–22 = 25–30; head length 27.9–33.0%; body depth 40.2–47.2%; eye diameter 35.5–45.5%; upper jaw length 50.0–56.7%; maximum 126 mm SL; scales weakly ctenoid, deciduous, thin, semicircular in shape, far wider than long (see Koeda et al. 2013a: fig. 2a); body golden in day time and silverish in night time; tip of dorsal fin and/or anterior margin of dorsal fin blackish; faint blackish band on anal-fin outer margin; blackish band on anal-fin base; posterior margin of caudal fin dusky; no blackish blotch on pectoral-fin base; posterior nostril usually slit-like; narrow band of villiform teeth in jaws; abdomen cross-sectional outline V-shaped.

#### Distribution.

Widely distributed in the western Pacific Ocean. In Japanese waters, this species is known from Tanega-shima to Yonaguni-jima islands in the Ryukyu Archipelago, Minamidaito-jima Island in Daito Islands, and very rarely collected from Minamisatsuma in Kagoshima Prefecture. In Taiwanese waters, this species is known from Daxi in Yilan County, Gungliau and Yeh Liu in New Taipei City, Chi-gu in Tainan County, Ke-tzu-liao in Kaohsiung County, Hengchung, and Dong-gang, Kenting in Pingtung County, Fugang in Taitung County, Lanyu, and Penghu (Fig. 13). Specimens are collected from the caves or crevasses in coral reef areas of 0–25 m depth.

#### Remarks.

*Pempherisschwenkii* has been thought to be widely distributed in the Indo-Pacific Ocean. However, our genetic study revealed the interspecific difference between specimens from the Indian and Pacific oceans, and southern Japan. Bleeker (1855) described *P.schwenkii* based on type specimens collected from Batu Island of western Indonesia, eastern Indian Ocean. Although a significant genetic difference is observed among the specimens from these three localities, the morphologies of the species are very similar. Furthermore, the morphological characters were difficult to determine from the dehydrated condition of the syntypes of *P.schwenkii* (RMNH.PISC.6160). The species composition of genus *Pempheris* around this area was closer to that of the western Pacific than that of the Indian Ocean. Therefore, *P.schwenkii* was determined as the name of the Pacific Ocean species (Koeda et al. 2014) and the Indian Ocean species described as a new species, *P.tominagai* Koeda, Yoshino & Tachihara, 2014. In the present study, a single specimen collected from the Andaman Sea was discovered, and is identified as *P.schwenkii* based on the pink caudal fin (vs yellow in *P.tominagai*). This fact supports the conclusion of Koeda et al. (2014) that *P.schwenkii* may be widely distributed from the Pacific to the Andaman Sea including the type locality (Batu Islands, Sumatera Utara Province, Indonesia) of the species.

Similarly, the Pacific species and the southern Japanese species also showed significant difference between specimens from south of the Ryukyu Archipelago and specimens from mainland Japan (unpublished data). These two are clearly different species, because both species are distributed in the Osumi Islands (Tanega-shima, Yaku-shima, and Kuchinoerabu-jima islands; Fig. 13), but the genetic mixability did not appear in the genetic structure analysis (unpublished data); the genetic identification and the diagnostic caudal-fin colorations (pink to brown in *P.schwenkii* vs yellow in *P.xanthoptera*) were well matched (Fig. 3).

On the basis of the taxonomic confusion between *P.schwenkii* and *P.xanthoptera*, the standard Japanese name “Minami-hatampo” was used for both species, and recently, *P.schwenkii* was tentatively recognized as having “Pacific” and “southern Japan” types, the latter closely matching *P.xanthoptera* sensu Tominaga (1963) (e.g., Koeda 2017b, 2018a, b, 2020a; Kimura et al. 2017); see remarks of *P.xanthoptera*]. The Japanese name “Minami-hatampo” was first given by Okada (1938) for *P.vanicolensis* in his list. His identification may follow Jordan and Hubbs (1925) which indicated that Snyder (1912)’s *P.japonica* from Okinawa-jima Island was a misidentification of *P.vanicolensis*. However, *P.vanicolensis* is very rare in Japanese waters, and has never been collected from Okinawa-jima Island (Nakamura et al. 2022). In addition, the re-examination of the Snyder’s specimen of *P.japonica* (CAS-SU 22002) revealed that it was in fact a misidentification of *P.schwenkii*, which is the most common species around Okinawa Island. Although Okada (1938) included Kyushu in the distribution of “Minami-hatampo” which is the range of *P.xanthoptera*, the situations mentioned above suggest that his species should be *P.schwenkii*. These facts indicate that the standard Japanese name “Minami-hatampo” should be adopted for the species *P.schwenkii*.

The juveniles of *P.schwenkii* were collected from Minami-daito Island in the Daito Islands. *Pempherisufuagari* is known as an endemic species which is found in the Daito and Ogasawara islands (see below), meaning that interaction between the species can occur at these localities. However, *P.schwenkii* and *P.xanthoptera* have never been collected from the Ogasawara and Daito islands, respectively, indicating that the *P.ufuagari* and *P.schwenkii* group (with *P.xanthoptera*) may have a different dispersal strategy.

### ﻿ Pempheris ufuagari

Taxon classificationAnimaliaPerciformesPempheridae

Koeda, Yoshino & Tachihara, 2013

3A60B638-6666-535A-82CA-6E59F048ECE1

**Figure 14.**
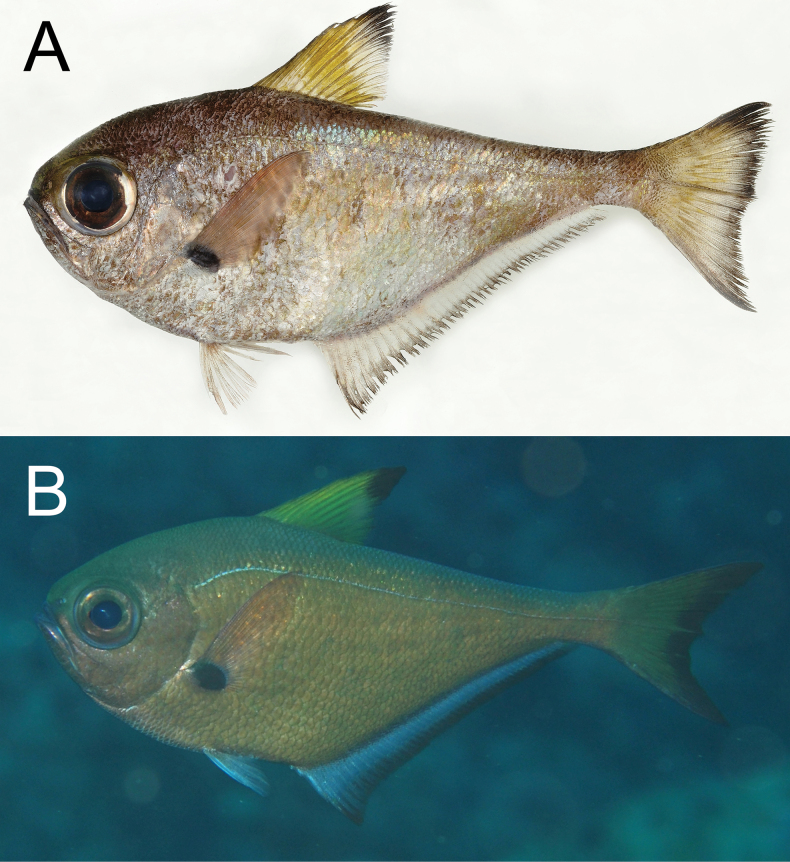
*Pempherisufuagari***A** fresh specimen (KAUM–I. 74550, Chichi-jima Island, Ogasawara Islands, 170.3 mm SL, photo taken by K. Kuriiwa) and **B** underwater photograph (Dobuiso, Ogasawara Islands, Japan).

**Figure 15.**
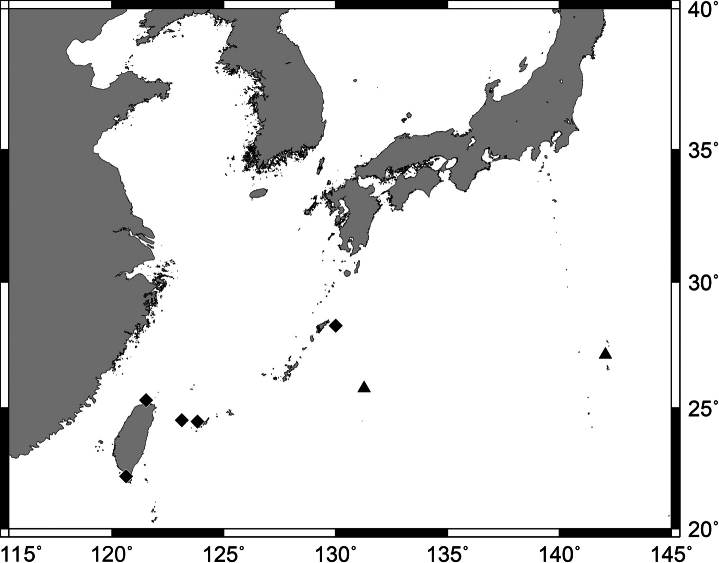
Distribution of *Pempherisufuagari* (triangles) and *P.vanicolensis* (diamonds) based on the collection localities of the specimens.

Figs 14 , 15 , Suppl. material 2 Standard Japanese name: Daito-hatampo

Pempheris ufuagari Koeda et al. 2013a: 232, fig. 1a (type locality: Minamidaito-jima Island, Daito Islands, Japan); Koeda and Motomura 2015: 275; Koeda 2017a: 11, fig. 2 (lower fig.); Koeda and Motomura 2017a; Koeda 2018b: 299, unnumbered fig.
Pempheris oualensis (not Cuvier, 1831): Masuda and Kobayashi 1994 (in part); 180, fig. 5; Hatooka 1997 (in part): 380, unnumbered fig. (p. 381, upper left fig.).
Pempheris otaitensis (not Cuvier, 1831): Randall et al. 1997 (in part): 35, pl. 8, fig. F.

#### Diagnosis.

Counts of holotype and paratypes are given in Table 1 of Koeda et al. (2013a). Dorsal-fin rays VI, 9; anal-fin rays III, 39–43; pectoral-fin rays 17–18; pored lateral-line scales 62–71; scale rows above lateral line 6½–7½ (usually 7½); scale rows below lateral line 15–18; predorsal scales 37–44; circumpeduncular scales 20–22; gill rakers 8–9 + 20–21 = 28–30; head length 26.2–29.9%; body depth 39.7–44.4%; eye diameter 37.5–45.8%; upper jaw length 48.8–53.4%; maximum 197 mm SL, usually < 170 mm SL; scales weakly ctenoid, deciduous, thin, semicircular in shape, far wider than long (Koeda et al. 2013a: see fig. 2a); body silver; distinct blackish blotch on pectoral-fin base; dorsal fin yellow with tip blackish; blackish band on anal-fin outer margin; caudal fin bright yellow with blackish posterior margin; lacking villiform tooth band extending outside lips; abdomen cross-sectional outline V-shaped.

#### Distribution.

Endemic to the Daito and Ogasawara islands (Fig. 15). Specimens collected from the crevasses with strong current in coral reef areas of 0–20 m depth.

#### Remarks.

*Pempherisufuagari* is most similar to *P.otaitensis*, known only from French Polynesia and Samoa, sharing a distinct black blotch on the pectoral-fin base, yellow dorsal and caudal fins, and a blackish band on the anal-fin outer margin. However, the former can clearly be distinguished from the latter in having 62–71 pored lateral-line scales (vs 69–79 in *P.otaitensis*), 6½–7½ scale rows above lateral line (vs 8½), 37–43 predorsal scales (vs 44–48), and the tip of the dorsal fin blackish (vs anterior margin to tip blackish).

### ﻿ Pempheris vanicolensis

Taxon classificationAnimaliaPerciformesPempheridae

Cuvier, 1831

5912D4E8-621D-5DB9-BF5E-91576B60D30B

**Figure 16.**
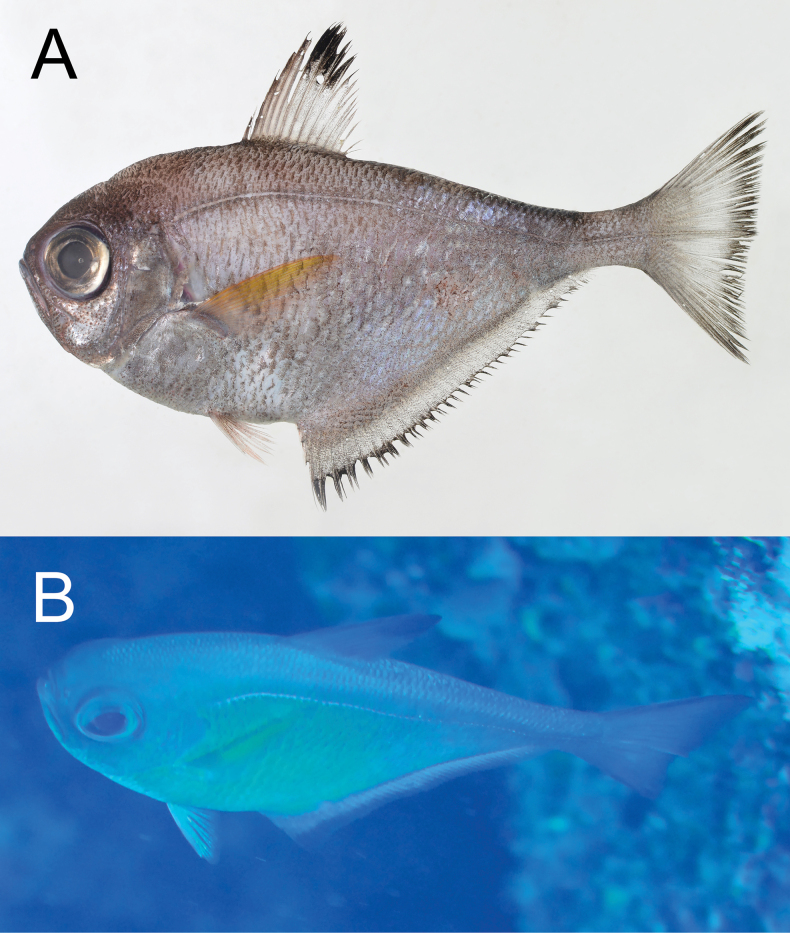
*Pempherisvanicolensis***A** fresh specimen (KAUM–I. 65386, 132.7 mm SL, Hengchung, Pingtung, Taiwan) and underwater photograph (Palau).

Figs 15 , 16 , Suppl. material 2 Standard Japanese name: Kibire-hatampo

Pempheris vanicolensis Cuvier, 1831: 305 (type locality: Vanikoro Island, Santa Cruz Islands); Shao and Chen 1991: 163, unnumbered fig.; Shao et al. 1992: 177, unnumbered fig.; Shen 1993: 391, pl. 114, fig. 2; Koeda et al. 2010b: 78, fig. 1; Koeda et al. 2012a: 71; Koeda et al. 2013a: 237; Koeda et al. 2013b: 127; Chiang et al. 2014: 183, unnumbered fig.; Koeda et al. 2014: 327; Koeda and Motomura 2015: 139; Koeda et al. 2015: 275; Koeda et al. 2016b: 50, fig. 226; Koeda 2017a: 5; Koeda 2018b: 299, unnumbered fig.; Koeda 2019: 929, unnumbered figs; Koeda et al. 2022: 11; Nakamura et al. 2022: 1, fig. 1.
Pempheris sp.: Shao et al. 2013 (in part): 161, unnumbered fig. (middle fig.); Shao et al. 2008: 254; Hatooka and Yagishita 2013 (in part): 984.

#### Diagnosis.

Counts of holotype and non-types are given in Table 2 of Koeda et al. (2010b). Dorsal-fin rays VI, 9; anal-fin rays III, 38–43; pectoral-fin rays 17–19; pored lateral-line scales 56–65; scale rows above lateral line 5½–6½, usually 5½; scale rows below lateral line 12–15; predorsal scales 30–35; circumpeduncular scales 16–18; gill rakers 8+19–21 = 27–29; head length 29.1–31.7%; body depth 42.4–46.3%; eye diameter 37.5–42.9%; upper jaw length 48.9–55.3%; maximum 156 mm SL; snout rounded; scales weakly ctenoid, deciduous, thin, semicircular in shape, far wider than long (Koeda et al. 2013a: see fig. 2a); body silverish to copperish in fresh specimen collected in day time, but silver in night time; tip of dorsal fin distinctly blackish; usually distinct blackish band on outer edge of anal fin; pectoral fin bright yellow (disappear in fixed specimens) with lacking blackish blotch on its base; posterior margin of caudal fin blackish; narrow band of villiform teeth in jaws; abdomen cross-sectional outline V-shaped.

#### Distribution.

Widely distributed in the western Pacific Ocean except for small islands and atolls in central Pacific. In Japanese waters, this species is known from Iriomote-jima and Yonaguni-jima islands in the southern Ryukyu Archipelago. In Taiwanese waters, this species is known from Yeh Liu and Wang-li in New Taipei City, and Hengchung and Kenting in Pingtung County (Fig. 15). Specimens were collected from 0–2 m depth in Japan but are known from deeper (ca 20 m) in other areas. KK observed a small school of this species at Kuchinoerabu-jima Island (24 Aug. 2016).

#### Remarks.

This species was described by Cuvier (1831), and the name *P.vanicolensis* has been used for several species, particularly from the Indian Ocean and the Red Sea. However, this species has only been collected from the Pacific Ocean and not from the Indian Ocean as shown in the present study. Jordan and Hubbs (1925) reported that the “*P.japonica*” documented by Snyder (1912) from Okinawa Island was a misidentification of *P.vanicolensis*. The early period of modern-day ichthyology in Japan probably followed Jordan and Hubbs (1952), and *P.vanicolensis* was recognized as “Minami-hatampo” in the species lists of Japanese waters (e.g., Okada 1938; Matsubara 1955). However, *P.vanicolensis* is very rare in Japan, and re-examination of Snyder’s specimen (SU 22002) revealed that it was a misidentification of *P.schwenkii*, which is the most common species around Okinawa Island.

### ﻿ Pempheris xanthoptera

Taxon classificationAnimaliaPerciformesPempheridae

Tominaga, 1963

B7992AB5-FDBC-548F-AAD0-E9E07B59971B

**Figure 17.**
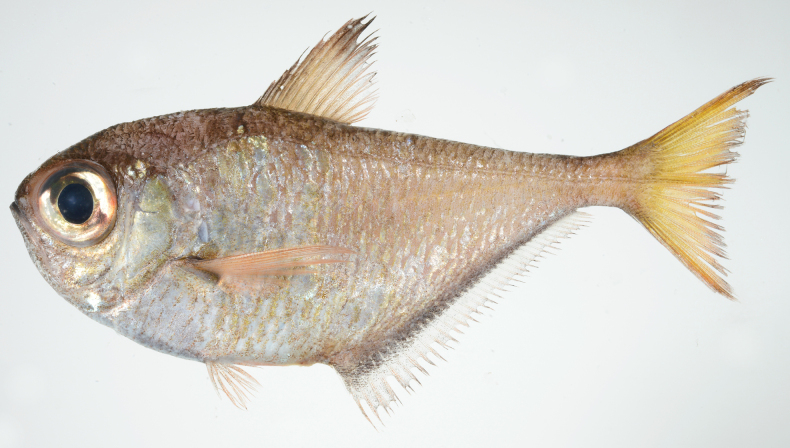
Fresh specimen of *Pempherisxanthoptera*, KBF-I 00268, 123.0 mm SL, Amaji, Otsuki, Kochi.

Figs 13 , 17 , Suppl. material 2 New standard Japanese name: Mizuho-hatampo

Pempheris xanthoptera Tominaga, 1963: 286, fig. 12 (type locality: Manazuri, Kanagawa Prefecture, Japan); Hiyama and Yasuda 1971: 204, fig. 440; Masuda et al. 1975: 199, pl. 33-C; Hayashi 1984: 160, pl. 151-D; Kohno 1986: 135, fig. 1; Randall et al. 1997: 35; Koeda et al. 2022: 11.
Pempheris molucca (not Cuvier, 1829): Temminck and Schlegel 1844: 85, pl. 44, fig. 3.
Pempheris oualensis (not Cuvier, 1831): Mochizuki 1995: 389, unnumbered fig. (in part).
Pempheris schwenkii (not Bleeker, 1855): Masuda and Kobayashi 1994: 180, fig. 6; Mochizuki 1995: 389, unnumbered fig.; Hatooka 1997 (in part): 380, unnumbered fig. (p. 381, lower left fig.); Hatooka 2002 (in part): 878; Takayama et al. 2003: 1317, fig. 2; Senou et al. 2006b: 463; Aramata 2007: 172, unnumbered figs; Yoshino 2008 (in part): 211; Takagi et al. 2010: 69, unnumbered figs; Kohno et al. 2011: 208, unnumbered fig.; Senou et al. 2012: 212; Hatooka and Yagishita 2013 (in part): 984; Koeda et al. 2013a: 237; Motomura et al. 2013 (in part): 168, unnumbered fig.; Kawano et al. 2014: 48; Koeda et al. 2014: 327; Ikeda and Nakabo 2015: 160, figs 1–3; Takeuchi et al. 2015: 8; Iwatsubo et al. 2016: 22, unnumbered figs; Kaburagi 2016: 98, lower fig. (without scientific name; indicated as “Minami-hatampo”); Kimura et al. 2017 (in part): 120, fig. 2; Koeda 2017a: 9, fig. 2 (middle); Koeda 2017b: 190, unnumbered fig.; Koeda and Motomura 2017a; Kagoshima City Aquarium Foundation 2018: 210, unnumbered fig.; Koeda 2018b: 298, unnumbered fig. (lower left fig.); Koeda 2018c: 343, unnumbered figs; Murase et al. 2019: 132, fig. 284; Koeda 2020a: 407, unnumbered figs; Murase et al. 2021: 166, fig. 340; Koeda 2022: 158, unnumbered fig.; Motomura 2023: 129 (in part).
Pempheris vanicolensis (not Cuvier, 1831): Okada 1938: 179 (in part); Okada and Matsubara 1938: 179 (in part); Matsubara 1955: 590 (in part); Abekawa and Nishi 1969: 24.
Pempheris japonica (not Döderlein, 1883): Nakamura 1993: 148, fig. 6.

#### Diagnosis.

Counts of of holotype and paratypes are given in Table 2. Dorsal-fin rays VI–VII, 9; anal-fin rays III, 35–42; pectoral-fin rays 16–19; pored lateral-line scales 45–54; scale rows above lateral line 3 1/2; scale rows below lateral line 10–13; predorsal scales 23–28; circumpeduncular scales 12–14; gill rakers 7–9+18–21 = 25–27; head length 27.9–31.9%; body depth 39.3–45.4%; eye diameter 36.4–44.1%; upper jaw length 48.3–57.1%; maximum 137 mm SL; scales weakly ctenoid, very deciduous, thin, semicircular in shape, far wider than long (see Koeda et al. 2013a: fig. 2a); body golden in daytime, but silver in night time; no blackish blotch on; tip of dorsal fin distinctly blackish; anal-fin base zonal blackish, and margin very faintly blackish; caudal fin yellow (disappears in fixed specimens); pectoral and pelvic fin hyaline or pink; posterior margin of caudal fin dusky; body light brown to dark brown in fixed specimen; black pigmentation on each fins usually persistent; no blackish blotch on pectoral-fin base; posterior nostril usually open, not compressed; narrow band of villiform teeth in jaws; abdomen cross-sectional outline V-shaped.

#### Distribution.

Endemic to the Northwest Pacific, recorded only from Japan, Jeju Island in Korea (Kim and Sakai 2004), and China. In Japanese waters, this species is known from Pacific coast (north to Boso Peninsula in Chiba Prefecture, south to Kagoshima Prefecture), Tsushima Island, East China Sea coast (north to Goto Islands in Nagasaki Prefecture, south to Kagoshima Prefecture), Izu-oshima, Miyake-jima and Hachijo-jima islands in Izu Islands, Chichi-jima, Haha-jima, Ototo-jima islands in Ogasawara Islands, Tanega-shima, Yaku-shima, Kuchinoerabu-jima, Iou-jima and Take-shima islands in northern Ryukyu Archipelago (Fig. 13).

#### Remarks.

Although Tominaga (1963) described *P.xanthoptera* based on its differences in fin color and distributional pattern from *P.schwenkii*, the former has been usually considered as a junior synonym of the latter in recent publications in Japan (see the synonym list) without any discussion. Our genetic analysis revealed apparent differences between these two species with high node support values (Fig. 3). The morphological comparison showed the additional small difference between these two species, such as *P.xanthoptera* has the posterior nostril usually open, not compressed (vs slit-like; see Koeda et al. 2014: fig. 8), and caudal fin yellow (vs pink to brown). The validity of *P.xanthoptera* was discussed in the remarks of *P.schwenkii*.

The standard Japanese name “Minami-hatampo” was used for both species and caused confusion, but this name should be adopted for *P.schwenkii* (see remarks of *P.schwenkii*). Therefore, a new standard Japanese “Mizuho-hatampo” is proposed for *P.xanthoptera*. “Mizuho” is an alternative name for Japan that frequently appears in ancient Japanese mythology and poetry, and it derives from the fact that the species is primarily distributed across the Japanese mainland.

## Supplementary Material

XML Treatment for Pempheris

XML Treatment for Pempheris adusta

XML Treatment for Pempheris familia

XML Treatment for Pempheris japonica

XML Treatment for Pempheris nyctereutes

XML Treatment for Pempheris oualensis

XML Treatment for Pempheris sasakii

XML Treatment for Pempheris schwenkii

XML Treatment for Pempheris ufuagari

XML Treatment for Pempheris vanicolensis

XML Treatment for Pempheris xanthoptera

## ﻿Additional information

### Author ORCIDs

Keita Koeda https://orcid.org/0000-0003-3932-3002

Manabu Bessho-Uehara https://orcid.org/0000-0002-7388-464X

### Data availability

All of the data that support the findings of this study are available in the main text or Supplementary Information.

## Supplementary materials

Supplementary material 1 Preserved specimens of genus *Pempheris* examined in the present study Data type xlsx https://binary.pensoft.net/file/1192481This dataset is made available under the Open Database License (http://opendatacommons.org/licenses/odbl/1.0/). The Open Database License (ODbL) is a license agreement intended to allow users to freely share, modify, and use this Dataset while maintaining this same freedom for others, provided that the original source and author(s) are credited.Keita Koeda, Manabu Bessho-Uehara

Supplementary material 2 requency distributions of pored lateral-line scales of species of *Pempheris* distributed in Japan and Taiwan, with *P.flavicycla* for comparison Data type xlsx https://binary.pensoft.net/file/1192482This dataset is made available under the Open Database License (http://opendatacommons.org/licenses/odbl/1.0/). The Open Database License (ODbL) is a license agreement intended to allow users to freely share, modify, and use this Dataset while maintaining this same freedom for others, provided that the original source and author(s) are credited.Keita Koeda, Manabu Bessho-Uehara
